# Dichotomic effects of clinically used drugs on tumor growth, bone remodeling and pain management

**DOI:** 10.1038/s41598-019-56622-5

**Published:** 2019-12-27

**Authors:** David André Barrière, Élora Midavaine, Louis Doré-Savard, Karyn Kirby, Luc Tremblay, Jean-François Beaudoin, Nicolas Beaudet, Jean-Michel Longpré, Roger Lecomte, Martin Lepage, Philippe Sarret

**Affiliations:** 1 Département de Pharmacologie-Physiologie/Institut de Pharmacologie de Sherbrooke, Université de Sherbrooke, Québec, Canada; 2 Département de médecine nucléaire et de radiobiologie, Université de Sherbrooke et Centre d’imagerie moléculaire de Sherbrooke, Sherbrooke, Québec, Canada; 3 Département d’Anesthésiologie, Université de Sherbrooke, Québec, Canada

**Keywords:** Bone cancer, Bone cancer

## Abstract

Improvements in the survival of breast cancer patients have led to the emergence of bone health and pain management as key aspects of patient’s quality of life. Here, we used a female rat MRMT-1 model of breast cancer-induced bone pain to compare the effects of three drugs used clinically morphine, nabilone and zoledronate on tumor progression, bone remodeling and pain relief. We found that chronic morphine reduced the mechanical hypersensitivity induced by the proliferation of the luminal B aggressive breast cancer cells in the tumor-bearing femur and prevented spinal neuronal and astrocyte activation. Using MTT cell viability assay and MRI coupled to ^18^FDG PET imaging followed by *ex vivo* 3D µCT, we further demonstrated that morphine did not directly exert tumor growth promoting or inhibiting effects on MRMT-1 cancer cells but induced detrimental effects on bone healing by disturbing the balance between bone formation and breakdown. In sharp contrast, both the FDA-approved bisphosphonate zoledronate and the synthetic cannabinoid nabilone prescribed as antiemetics to patients receiving chemotherapy were effective in limiting the osteolytic bone destruction, thus preserving the bone architecture. The protective effect of nabilone on bone metabolism was further accompanied by a direct inhibition of tumor growth. As opposed to zoledronate, nabilone was however not able to manage bone tumor-induced pain and reactive gliosis. Altogether, our results revealed that morphine, nabilone and zoledronate exert disparate effects on tumor growth, bone metabolism and pain control. These findings also support the use of nabilone as an adjuvant therapy for bone metastases.

## Introduction

The heterogeneity among breast cancers represents a major challenge for the diagnosis, prognosis, and sensitivity to drug treatment^1,2^. Of great importance in the prognosis of breast cancer patients is the sequence of events leading to the spread of cancer cells from the primary tumor to distant sites, the 5-year survival rate decreasing drastically from 95% in early-diagnosed patients to 30% in metastasis-bearing patients^3^. Breast cancer has a great propensity to metastasize to the skeleton, especially to the spine and long bones^4^. When breast cancer cells colonize the bones, they induce osteolysis leading to hypercalcemia, spontaneous fractures and debilitating pain^5^. Indeed, cancer cells induce sprouting and reorganization of sensory nerve fibers within the bone as well as osteoclastogenesis, therefore exposing free nerve terminal endings and promoting microfractures. Tumor expansion in the medullary channel also compresses bone nociceptors and induces stretching of the densely innerved periosteum. These biomechanical forces applied to the weakened cancer-bearing bone therefore lead to debilitating breakthrough pain episodes. Moreover, tumor cells release an array of cytokines, which promotes inflammatory processes, tumor growth, nociceptor sensitization and pain^6,7^. Hence, the combination of neuropathic and inflammatory processes combined with skeletal-related events are responsible for the unique profile of bone cancer pain, which often remains intractable^5,8–11^.

Clinically, cancer patients with bone metastases receive morphine to alleviate moderate to severe pain. However, there is currently debate in the literature regarding possible promoting effect of morphine on tumor progression^12,13^. Morphine is known to act on non-neural cells, including tumor and immune cells^14,15^. Accordingly, several studies have demonstrated that morphine can inhibit the growth of various human cancer cell lines, including breast cancers, while others have showed tumor-promoting effects of morphine in breast and osteosarcoma cancer models^16^. Importantly, previous findings also reported that opioids could directly disrupt bone homeostasis and facilitate osteoporosis and bone fractures^16–20^. Consequently, treating bone metastasis-bearing patients with morphine may have deleterious effects on the disease progression.

In patients coping with bone metastasis, bisphosphonate treatment is part of the conventional therapy^21^. Since 65–75% of patients with advanced breast cancer are typically at risk of developing bone metastases throughout their disease course^3^, zoledronate, being the most commonly used bisphosphonate is recommended to prevent or delay skeletal-related events in early breast cancer patients^22,23^. Bisphosphonates are synthetic and chemically stable analogs of pyrophosphates possessing high affinity for the bone matrix and acting as effective inhibitors of osteoclast-mediated bone resorption^24^. In addition to their antiresorptive action, bisphosphonates have also been described in preclinical studies to exert indirect or even direct anticancer properties^25,26^. Large randomized controlled clinical trials also support the anti-tumor activity of adjuvant bisphosphonates (especially zoledronate) in patients with early-stage breast cancer, the potential benefits relating to decrease in distant metastases, fracture risk reduction and increased disease-free survival^27,28^. As for morphine, there are, however, some safety concerns about prolonged use of bisphosphonates. Indeed, patients taking zoledronate are at risk of developing osteonecrosis of the jaws, cardiovascular toxicity and impairment of renal function^29^.

Among the potential therapeutic targets, cannabinoids have emerged as promising options for the treatment of tumor-induced bone loss as well as for the management of pain. Accordingly, a number of recent preclinical studies ranging from *in vitro* studies, xenografts to genetically engineered mice support the use of cannabinoids as adjuvant agents to conventional anticancer therapies^30–32^. Likewise, there is increasing evidence demonstrating that the endocannabinoid system plays an important role in bone homeostasis^33,34^. The development of cannabinoid-based medications also shows a great promise for the treatment of pain. Despite the lack of robust clinical findings, cannabinoids acting on both cannabinoid receptor type 1 (CB1) and 2 (CB2) have, indeed, been found to exhibit antinociceptive properties in preclinical models of neuropathic, inflammatory and cancer pain^35,36^. Among the cannabinoids approved for medical purposes, nabilone (Cesamet), a dual CB1/CB2 receptor agonist, which is a synthetic analog of THC (Delta-9-Tetrahydrocannabinol), has received a growing interest during the last decade^37^. Indicated for the relief of chemotherapy-induced nausea and vomiting as well as for the treatment of patients with cancer-related anorexia-cachexia syndrome, nabilone is also emerging for its analgesic benefits among patients suffering from neuropathic or cancer pain^38,39^.

Considering that distant metastases are responsible for the great majority of deaths in breast cancer patients, there is an urgent need to develop new strategies that reduce skeletal tumor burden, prevent bone resorption, and achieve bone cancer pain control. In the present study, we therefore used a female rat model of syngeneic mammary rat metastasis tumor (MRMT-1) breast cancer-induced bone pain to study and compare the effects of chronic morphine or nabilone regimens and acute zoledronate administration on bone metastasis progression, bone remodeling and pain management.

## Results

### Characterization of the MRMT-1 rat breast carcinoma cells

**Figure 1:**
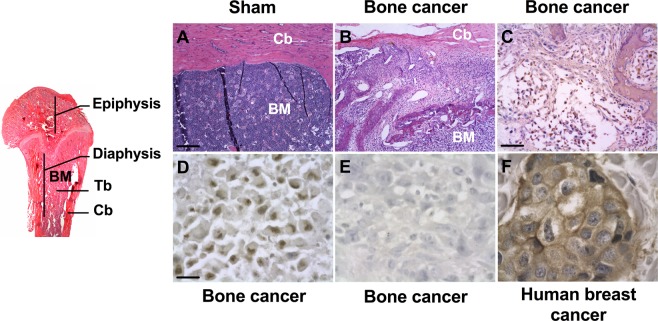
Histopathological and molecular characteristics of bone metastases in breast cancer, following intra-femoral inoculation of syngeneic MRMT-1 tumor cells. (**A**) Sham-operated female rats display a healthy femoral bone marrow (BM) and cortical bone (Cb) while (**B**) tumor-bearing rats exhibit intense cancer cell invasion into the bone marrow, 18 days following cancer cell implantation. (**C**) Extensive Ki-67-positive staining (brown-stained cancer cell nuclei) indicates a highly proliferative tumor, also expressing ERα (estrogen alpha receptor) (**D**) but not the HER-2 receptor (**E**). An anonymous human HER-2 breast cancer sample is used here as a positive control for HER-2 staining (**F**). Scale bars represent 100 µm in (**A**,**B**), 200 µm in (**C**) and 50 µm in (**D–F**).

Taking into account current clinical practice, we first evaluated the histological and molecular features of the MRMT-1 rat mammary tumor cells metastasizing to the femoral bone *in vivo*. Eighteen days after cancer cell implantation, severe damage to both cortical and trabecular bone were observed (Fig. 1B). This bony destruction was accompanied by a progressive disappearance of the bone marrow, with tumor cells invading nearby healthy tissue. In contrast, sham femurs displayed intact structure with healthy bone marrow and high trabecular and cortical densities (Fig. 1A).

Immunohistological staining is routinely used in breast cancer subtype classification and has demonstrated its potential for guiding oncologic prognosis and determining response to therapy. We therefore characterized the tumor cells within the bone environment with the most common immunohistochemical breast cancer markers used in pathology. The extensive staining observed with the Ki-67 proliferation marker reveals a high proliferation pattern of bone metastases derived from the MRMT-1 breast cancer cells (Fig. 1C). Immunohistochemistry staining on adjacent breast tumor tissue sections also revealed that the α-estrogen receptor (ERα, Fig. 1D) but not the HER2 receptor (Fig. 1E) was expressed *in vivo* by MRMT-1 cancer cells. The validity of our HER2 labeling procedure was assessed using an anonymous human HER2 breast cancer sample where an intense HER2-positive labeling was observed (Fig. 1F). Therefore, the osteolytic MRMT-1 breast-derived bone metastasis consists of ERα (+), HER2 (−), Ki-67 (+) tumor cells, which can be classified as a luminal B invasive adenocarcinoma^40,41^.

**Figure 2:**
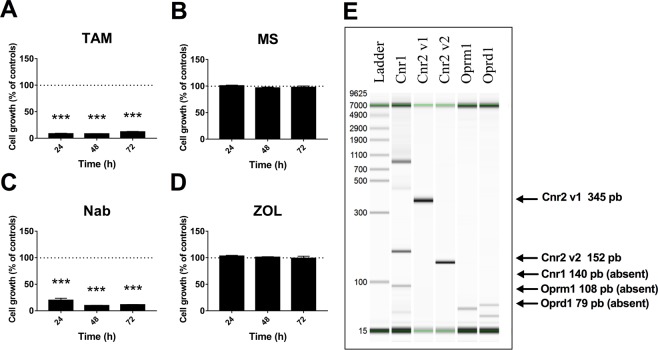
*In vitro* MRMT-1 cell viability upon 24 h, 48 h and 72 h of treatment with (**A**) tamoxifen, (**B**) morphine sulfate, (**C**) nabilone or (**D**) zoledronate. Tamoxifen (40 µM) and nabilone (10 µM) reduce MRMT-1 cell viability while treatment with either morphine sulfate (10 µM) or zoledronate (10 µM) fails to achieve such effect. (**E)** Quantitative RT-PCR analyses of the cannabinoid receptors CB1 (Cnr1) and CB2 (Cnr2), opioid receptors µ (Oprm1) and δ (Oprd1) expression in MRMT-1 cancer cells. Breast cancer cells are positive for both transcript variants of the CB2 receptor but are negative for CB1, µ- and δ-opioid receptors. Bars represent mean ± SEM, *n* = 3. ***p < 0.001 compared to control.

The presence of the α-estrogen receptor in MRMT-1 cancer cells indicates that this luminal lineage could be sensitive to tamoxifen. Accordingly, we observed *in vitro* that incubation of these cells with tamoxifen (TAM) significantly decreased cell growth after 24 h, 48 h and 72 h of treatment (Fig. 2A and Supplemental Fig. S1). We next investigated whether the morphine sulfate (MS) and nabilone (NAB) regimens or zoledronate (ZOL) treatment promoted or prevented the tumor cell growth. As demonstrated using the MTT cell viability assay, application of NAB significantly decreased MRMT-1 cell viability while MS and ZOL did not have any effect even following prolonged drug exposure (Fig. 2B–D and Supplemental Fig. S1). We also found using real-time quantitative PCR that the CB2 receptor was expressed by MRMT-1 cells while CB1 as well as µ- and δ-opioid receptors were absent (Fig. 2E and Supplemental Fig. S2). Altogether, these results support the idea that NAB may exert its antiproliferative action on the MRMT-1 mammary tumor cells via CB2. The lack of effect of MS on the MRMT-1 cell viability also correlates with the absence of the µ- and δ-opioid receptors.

### Monitoring tumor growth and treatment response by combining PET with MR imaging

**Figure 3:**
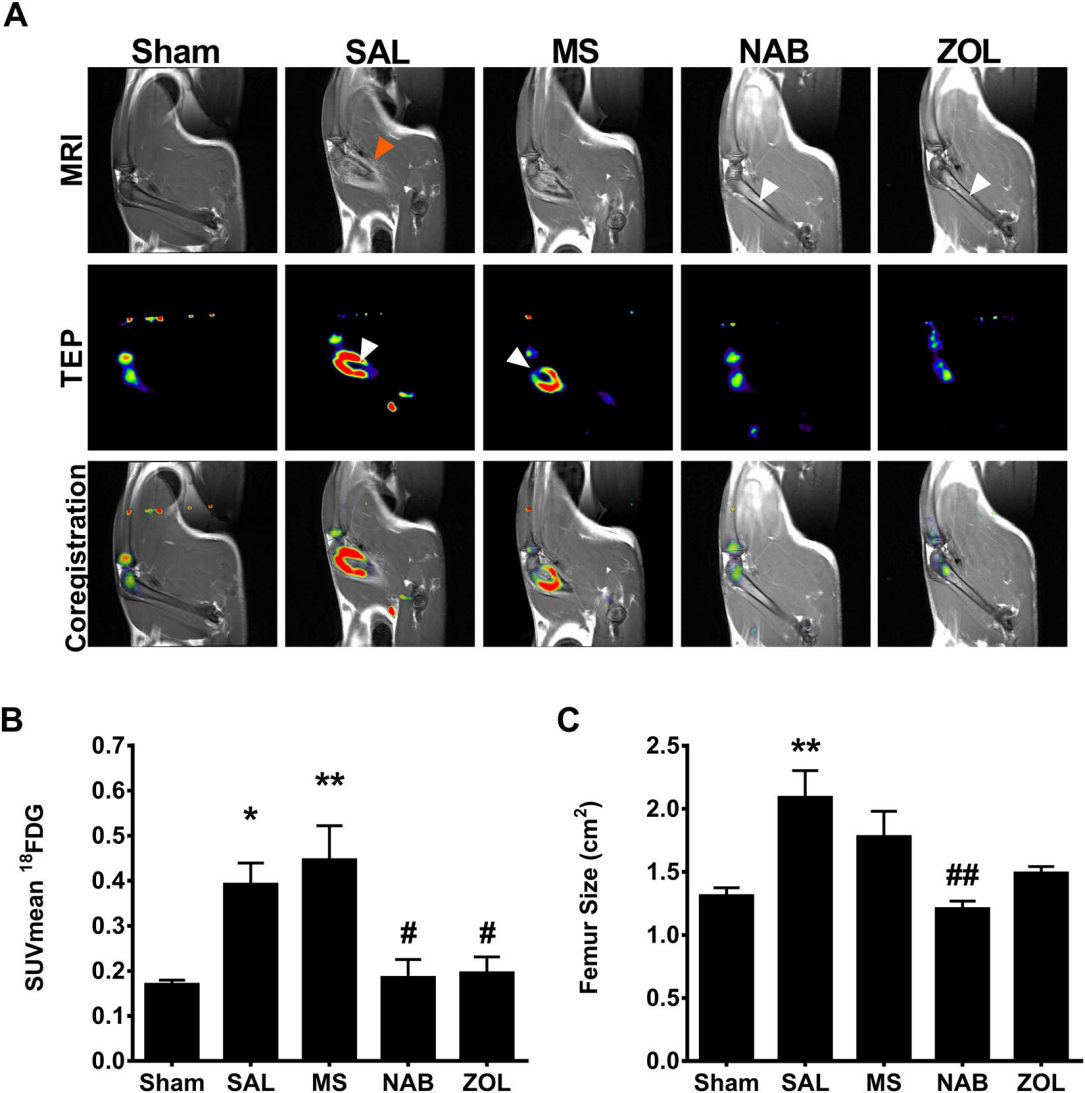
Effect of chronic morphine and nabilone regimens and bisphosphonate treatment on bone tumor metabolism, at day 18 post-cancer cell implantation. (**A**) In saline- and morphine-treated cancer-bearing animals, contrast-enhanced MRI images show that the tumor spreads into the distal medullar channel, inducing periosteal distention and bone epiphysis enlargement. Extraosseous inflammation is also observed by the presence of hyperintense signal in the vicinity of the femoral bone (orange arrowhead). In contrast, the breast cancer cells remain confined to the femoral medullary cavity of nabilone- and zoledronate-treated cancer-bearing rats. Also note that the cortical line exhibits a well-defined regular appearance (white arrows). ^18^F-FDG PET scans show a large ring-shaped region of ^18^F-FDG uptake in saline- and morphine-treated animals but not in nabilone and zoledronate groups. The inactive metabolic zone detected in the middle of the radiotracer hot spot corresponds to a necrotic region (white arrowheads). Co-registered images show strong co-localization of ^18^F-FDG uptake with the contrast-enhanced MR imaging in both saline and morphine groups. (**B**) Standardized uptake value (SUV_mean_) measurement determined by ^18^F-FDG PET. (**C**) Tumor area assessed on MRI scans at day 18 shows that bi-daily treatment for 7 days with saline or morphine increases femur size while these parameters remain unchanged in nabilone- and zoledronate-treated rats. Bars represent mean ± SEM, *n* = 5–6 animals per treatment group. *p < 0.05, **p < 0.01, ***p < 0.001 compared to sham animals and ^#^*p* < 0.05, ^##^*p* < 0.01, ^###^*p* < 0.001 compared to saline-treated rats.

The tumor development and structural damages to the femoral bone were monitored by magnetic resonance imaging (MRI) coupled to ^18^F-FDG-positron emission tomography (PET), this whole-body dual MRI/PET procedure providing accurate anatomical and functional information. We previously characterized this bone cancer pain model by monitoring the spatiotemporal tumor progression and bone remodeling over 21 days following femoral mammary carcinoma MRMT-1 cell implantation^8–10^. Here, we used the PET/MR image co-registration approach to determine the effects of MS and NAB chronic regimens on tumor proliferation and bone invasion. On day 18 after breast cancer cell inoculation, the extent of the tumor spread was compared to saline- (SAL) and ZOL-treated groups, the latter being the reference treatment for bone metastases. Contrast-enhanced MRI scans revealed the presence of a T1 signal hyperintensity throughout the femoral shaft of SAL- and MS-treated animals compared to the sham group (Fig. 3A). The intraosseous growth of the breast cancer cells was accompanied by enlargement of the epiphysis, extensive bone matrix destruction, and periosteal inflammation. Accordingly, ^18^F-FDG PET imaging showed a large uptake of the radiolabeled tracer in the diaphysis/metaphysis region in both SAL and MS groups (Fig. 3A). After drawing selected regions of interest (ROI) on the bone tissue, we found that the mean standardized uptake values (SUV_mean)_ was significantly increased in SAL- and MS-treated animals compared to sham (Fig. 3B). The tumor area assessed on the MRI scans on day 18 also revealed that the femur size increased in SAL- and MS-treated rats compared with that of sham-operated controls (Fig. 3C).

In sharp contrast, cancer-bearing rats treated chronically with NAB or acute ZOL exhibited a restricted expansion of the tumor cells within the medullary cavity, with no apparent effect on the surrounding bone and tissue environment (Fig. 3A). Co-registered ^18^F-FDG PET/MR images further revealed that the radiotracer accumulation was strongly reduced in the NAB- and ZOL-treated groups, thus indicative of a limited tumor progression. On the TEP scans, we further found that the SUV_mean_ was significantly decreased in NAB- and ZOL-treated groups compared to SAL-injected rats (Fig. 3B). On the other hand, NAB- and ZOL-treated animals both exhibited limited changes in femur size compared to SAL-injected control rats (Fig. 3C). Hence, we demonstrate that a chronic regimen of NAB or an acute ZOL administration limits the progression of the bone metastasis *in vivo* while chronic MS treatment is not effective to block tumor growth and spread.

### Bone resorption and remodeling assessed by µCT and histology in response to morphine, nabilone or zoledronate treatment

**Figure 4:**
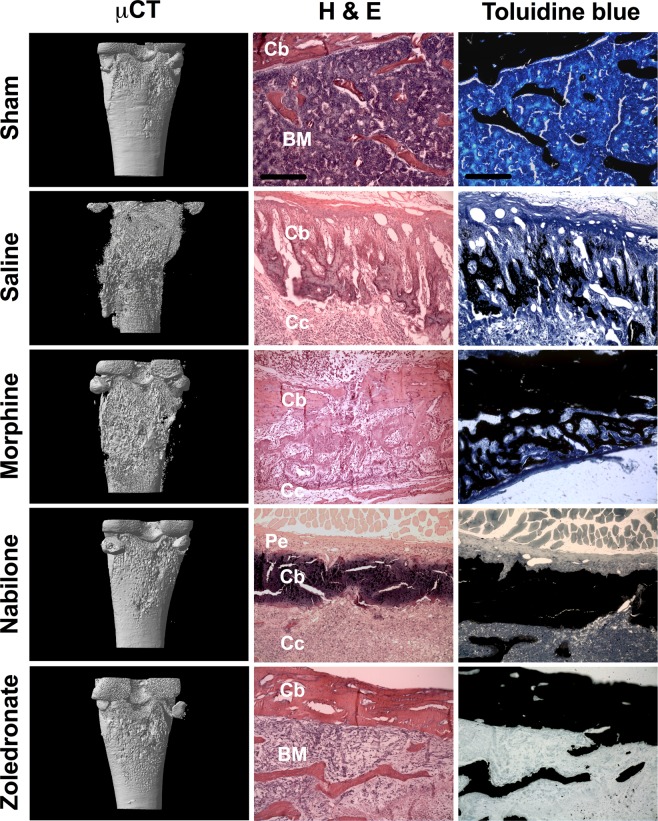
Effect of chronic morphine and nabilone regimens and bisphosphonate treatment on bone remodeling at day 18 post-cancer cell inoculation. µCT analyses reveal that the bone architecture is strongly affected in saline- and morphine-treated cancer-bearing rats compared to sham animals. The disorganization of the bone tissue was confirmed by H&E and Toluidine blue stainings. The µCT analyses and histological observations demonstrate that both nabilone and zoledronate are effective in reducing bone remodeling. In zoledronate-treated rats, we can observe that the cancer cells remain confined to the medullary cavity. BM: bone marrow; Cb: cortical bone; Cc: cancer cells; Pe: periosteum. Scale bar represents 100 µm.

**Figure 5:**
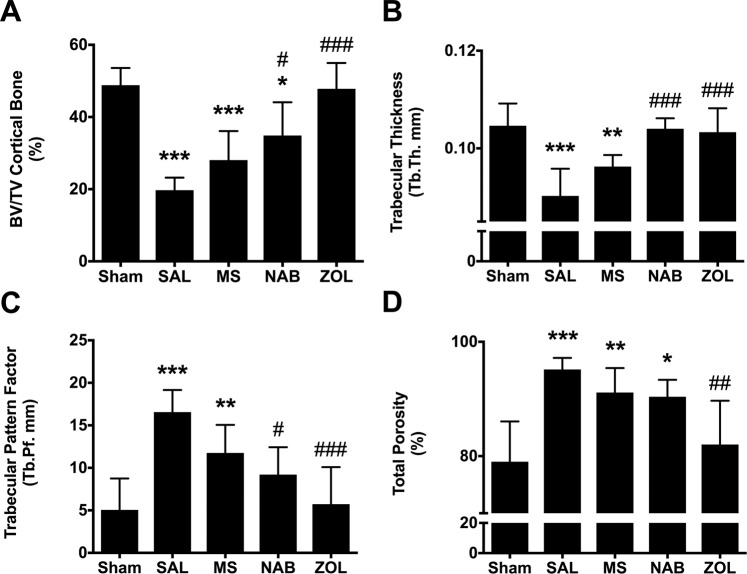
Effect of chronic morphine and nabilone regimens and acute bisphosphonate treatment on tumor-bearing bone degradation parameters. At day 18, µCT analysis reveals that BV/TV ratio (**A**) and trabecular thickness (Tb.Th; **B**) are significantly decreased in cancer-bearing saline- and morphine-treated animals, compared to shams. Conversely, both trabecular pattern factor (Tb.Pf; **C**) and cortical bone porosity (**D**) are increased. BV/TV ratio, trabecular thickness and trabecular pattern factor were decreased in nabilone- and zoledronate-treated animals compared to SAL-treated animals. Bars represent the mean ± SEM, *n* = 4–6 per treatment group. *p < 0.05, **p < 0.01, ***p < 0.001 as compared to sham and ^#^*p* < 0.05, ^##^*p* < 0.01, ^###^*p* < 0.001 as compared to saline-treated tumor-bearing rats.

The presence of metastatic breast cancer cells into the femur disturbs the bone microenvironment. Here, we further investigated the effect of MS or NAB regimen and bisphosphonate treatment on bone remodeling using post-mortem microcomputed tomodensitometry (µCT) imaging and histology. As opposed to the intact bone architecture of sham rats, 3D-µCT scans showed major bone destruction on day 18 in SAL- and MS-treated rats. Consistently, visual examination of the hematoxylin/eosin staining (H&E) and toluidine blue stainings, used as histological markers, highlighted that the tumor progression induced periosteal distension, large *lacunae* formation, and cortical bone destruction (Fig. 4). The bone loss was further confirmed by the measurement and quantification of different morphological parameters (Fig. 5). In SAL and MS groups, µCT scan analysis revealed that both cortical bone volume ratio, bone volume/tissue volume (BV/TV), and trabecular thickness (Tb.Th) were significantly decreased compared to the sham group (Fig. 5A,B, respectively). Moreover, the trabecular bone pattern factor (Tb.Pf), used as an index of trabecular connectivity and the cortical bone porosity assessing the overall bone strength were significantly increased in SAL- and MS-treated tumor-bearing rats (Fig. 5C,D, respectively).

In NAB-treated animals, we only observed a slight bone loss and remodeling. Tumor cells invading the femoral channel were found at the interface between cortical bone and periosteum but did not induce cortical bone remodeling as shown by 3D-μCT and confirmed by an intense toluidine blue calcium staining (Fig. 4). Furthermore, µCT analysis revealed that the BV/TV ratio, trabecular thickness and trabecular pattern factor were significantly different compared to SAL-treated tumor-bearing animals (Fig. 5A–C). The NAB treatment was, however, unable to reduce cortical bone porosity (Fig. 5D). In ZOL-treated animals, both cortical and trabecular bone remained unmodified and necrotic tumor cells were observed by histology inside the medullar cavity (Fig. 4). Importantly, all morphological bone parameters were found similar to sham-operated rats (Fig. 5). These data thus provide evidence that the chronic NAB regimen or acute ZOL administration efficiently prevents bone resorption induced by the homing of tumor cells to the bone while chronic MS treatment does not preserve the structural integrity of the bone.

**Figure 6:**
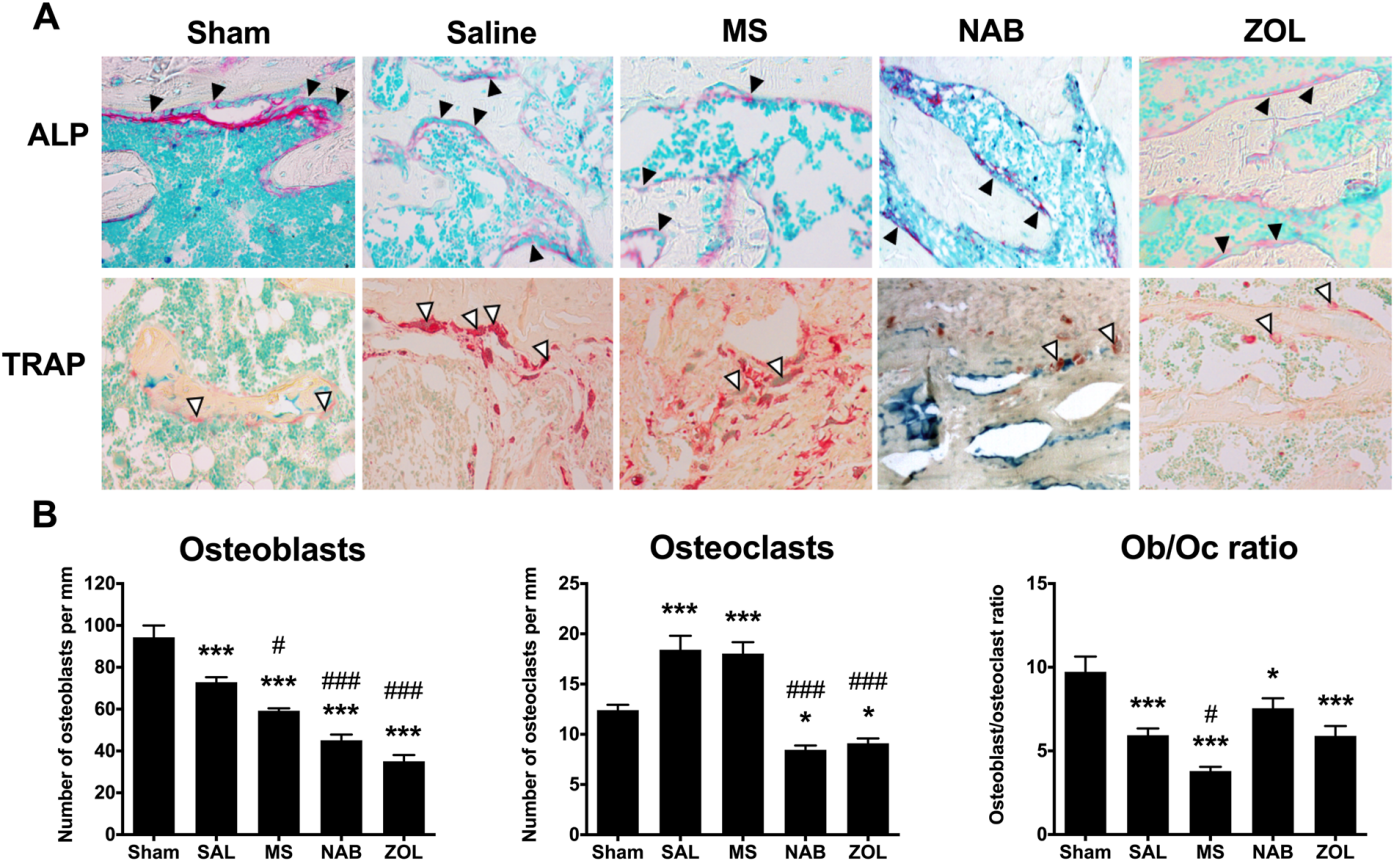
Effect of chronic morphine and nabilone regimens and acute bisphosphonate treatment on the osteoclast/osteoblast cell density in the tumor-bearing femur. (**A**) Alkaline phosphatase (ALP; top panel) and Tartrate-resistant acid phosphatase (TRAP; bottom panel) stainings were used to investigate osteoblast (black head arrows) and osteoclast (white head arrows) activity in the tumor-bearing femur. (**B**) At day 18, the number of osteoblasts, as determined by ALP activity, is decreased in all cancer-bearing animals. Chronic bi-daily treatment with morphine or nabilone, and zoledronate acute administration further reduce the number of osteoblasts as compared to saline-treated cancer-bearing rats. Both saline and morphine administration increase the osteoclast density, as determined by TRAP staining. In contrast, osteoclasts decrease significantly in number following nabilone and zoledronate treatment. Determination of the osteoblast/osteoclast ratio. Bars represent the mean ± SEM, *n* = 4–6 animals per treatment. *p < 0.05, **p < 0.01, ***p < 0.001 as compared to the sham group and ^#^*p* < 0.05, ^##^*p* < 0.01, ^###^*p* < 0.001 as compared to saline-treated tumor-bearing rats.

The presence of tumor cells in the bone marrow cavity disturbs the balance between bone resorption and bone formation, resulting in abnormal bone remodeling. To understand the protective effect of NAB and ZOL on bone metastasis progression, we evaluated the balance between bone-depositing (i.e. osteoblasts) and bone-degrading (i.e. osteoclasts) cells in the tumor-bearing femur. We used Alkaline phosphatase (AP) and Tartrate-resistant acid phosphatase (TRAP) labeling to quantify osteoblasts and osteoclasts in tumor-afflicted femurs (Fig. 6A). At the microstructural level, we observed a significant decrease in osteoblast number in all cancer-bearing rats as compared to sham animals, this decrease being more important in MS-, NAB- and ZOL-treated groups (Fig. 6B). In contrast, the number of osteoclasts significantly increased in SAL and MS groups compared to sham animals whereas NAB or ZOL treatment limited the osteoclast activity. Finally, the osteoblast/osteoclast ratio was decreased in all cancer-bearing animals compared to sham, highlighting that the bone homeostasis is greatly altered by breast cancer cell proliferation (Fig. 6B). MS was notably able to significantly increase bone catabolism compared to the SAL-treated group. In conclusion, MS decreased the osteoblast activity without affecting tumor-induced increase in osteoclast number compared to SAL-treated rats therefore increasing the production of lytic bone lesions and the risk of bone fractures. In contrast, NAB and ZOL were found to decrease the activity of both bone cell populations.

### Impact of morphine, nabilone and zoledronate treatment on the pain-related behaviors and spinal neuronal and glial activation

**Figure 7:**
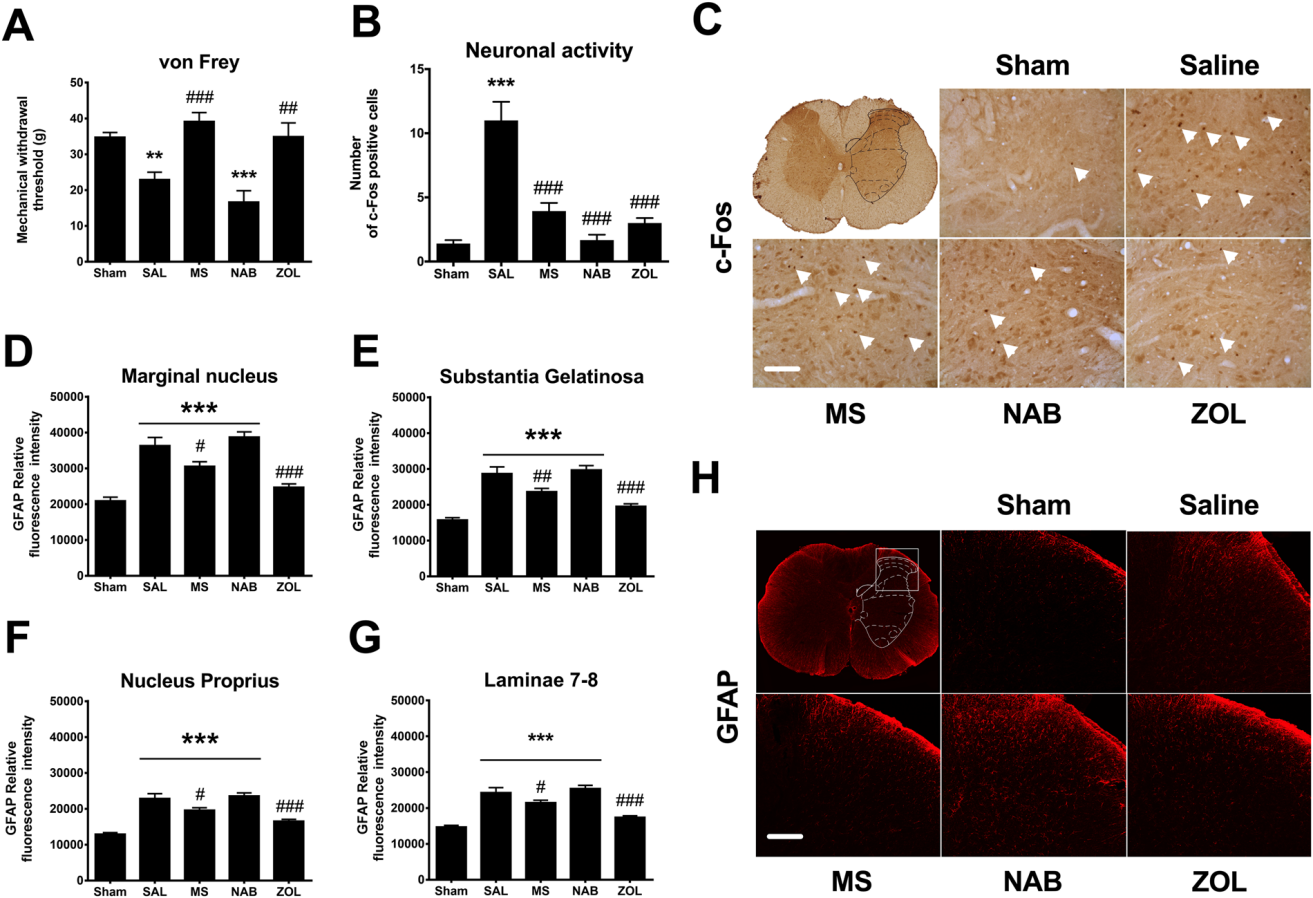
Effect of chronic morphine and nabilone regimens and acute bisphosphonate treatment on mechanical pain threshold and spinal sensitization. (**A**) At day 18 following cancer cell implantation, chronic MS administration and ZOL treatment increase mechanical pain threshold as compared to saline-injected rats while chronic nabilone treatment fails to alleviate bone cancer pain. (**B**) Number of c-Fos-positive cells is significantly increased in saline-injected rats while all three drugs effectively reduce neuronal activation in the spinal cord. (**C**) Expression of c-Fos protein in neurons of the spinal cord of bone cancer-bearing animals 18 days after implantation into the femur. White arrowheads point to c-Fos proteins only in the ipsilateral limb. GFAP labeling is increased in marginal nucleus (**D**), *substantia gelatinosa* (**E**), *nucleus proprius* (**F**) and *laminæ* 7–8 (**G**) in saline-, morphine- and nabilone-treated rats, compared to sham. However, morphine and zoledronate induce a reduction in astrocyte reactivity, compared to the saline group. (**H**) GFAP staining in dorsal horn of the L3 spinal cord of rats at day 18 post-surgery. Bars represent the mean ± SEM. *p < 0.05, **p < 0.01, ***p < 0.001 compared to sham rats and ^#^*p* < 0.05, ^##^*p* < 0.01, ^###^*p* < 0.001 when compared with the saline group. (n = 10 slices analyzed per condition with n = 3 rats per condition).

We next determined the therapeutic efficacy of chronic MS and NAB or acute ZOL for the management of bone cancer pain. At day 18 post-surgery, mechanical pain thresholds were significantly decreased in SAL-treated rats compared to sham animals. MS and ZOL treatments increased mechanical nociceptive thresholds to sham values, while NAB failed to alleviate mechanical allodynia (Fig. 7A). We next investigated the effect of MS, NAB and ZOL treatment on central sensitization. To this aim, immunohistochemical techniques were used to measure the changes in neuronal (c-Fos) and astrocyte (GFAP) activation at the spinal dorsal horn level. We found that the increase in number of c-Fos immunoreactive neurons observed in SAL-injected rats was significantly reduced by the treatment with MS, NAB or ZOL (Fig. 7B,C). Cancer-bearing rats also displayed significant increase in glial fibrillary acidic protein (GFAP) staining in the marginal nucleus, *substantia gelatinosa*, *nucleus proprius* and *laminæ* 7–8, as compared to sham animals (Fig. 7D–H). Importantly, MS and ZOL significantly reduced the GFAP immunolabeling in all spinal regions studied, whereas NAB was ineffective in reversing spinal astrocyte activation. Altogether, these data demonstrate that both MS and ZOL are effective analgesics for the treatment of painful osseous metastases, preventing spinal neuronal and astrocyte activation. Despite its effectiveness in blocking neuronal hyperactivity, chronic NAB treatment did not effectively manage the mechanical hypersensitivity nor limit the reactive astrogliosis.

## Discussion

The introduction and optimization of multimodal therapies and interdisciplinary approaches have considerably increased the survival rates of breast cancer patients. However, this success in treating primary tumors has given way to new challenges arising from the development of skeletal complications associated with bone metastases, including tumor-induced pain and increase in bone fractures. In recent decades, multiple preclinical models of bone cancer pain have been developed to faithfully mimic the human condition^42^. While the etiology of tumor-induced bone pain remains to be fully elucidated, these animal models have allowed greater understanding of the molecular and cellular mechanisms driving the development of painful bone metastases and have revealed the unique neurochemical signature underpinning bone cancer pain. Today, these preclinical models represent valuable tools to screen and optimize drug candidates as well as to validate their effectiveness in alleviating bone cancer pain, which severely compromises patients’ quality of life.

Bone cancer pain is a complex and multifaceted process involving numerous actors interacting to promote a vicious circle where bone, tumor, and immune cells as well as nociceptor terminals interplay to dramatically increase tumor growth, skeletal remodeling and debilitating pain. In the current study, we combined different medical imaging modalities with behavioral, histological and cell viability approaches to examine the impact of prescribed medications on bone health, tumor burden and pain relief in a clinically relevant, fully characterized syngeneic breast cancer bone metastasis pain model in female rats. Our results reveal that chronic treatment with morphine or nabilone and acute administration of zoledronate have disparate effects on tumor growth, affect differently the bone metabolism and give rise to different pain management outcomes.

### Histological, molecular and pharmacological characterization of the MRMT-1 breast carcinoma cells metastasizing to the femoral bone

Breast cancer is an extremely heterogeneous disease at both inter- and intra-tumoral levels and improvements in diagnosis, treatment, and prognosis require a refined molecular taxonomy of breast carcinoma. According to the St Gallen 2013 guidelines^43^, invasive breast cancer can be classified into five distinct molecular subtypes: luminal A-like (ER+, HER2−, Ki-67 low and PR high), luminal B-like HER2-negative (ER+, HER2−, and either Ki-67 high or PR low), luminal B-like HER2-positive (ER+, PR−, HER2+), HER2-positive (non-luminal) (ER−, PR−, HER2+), and triple-negative (ER−, PR−, HER2−). In clinical practices, this breast cancer subtype classification represents a useful prediction tool to identify patients with high risk of developing bone metastases^44^. Accordingly, oncology studies demonstrated that patients with a HER2-negative hormone receptor-positive (ER+ or PR+) status are more prone to bone metastases^45^ Here, we provided new features of the MRMT-1 tumor cells used to model metastatic bone pain, reporting that MRMT-1 rat breast carcinoma cells are ER-positive and HER2-negative with a high Ki-67 expression and a tamoxifen sensitive profile. This high proliferative activity, as revealed by Ki-67 staining is correlated with aggressive breast cancer, early recurrence and poor prognosis^46^. MRMT-1 cells do not express µ- and δ-opioid receptors, nor the CB1 receptor but do express CB2 receptor and are insensitive to morphine and zoledronate *in vitro*. These data thus provide a detailed characterization of the MRMT-1 lineage as a derived luminal B, HER2-negative invasive breast adenocarcinoma suitable to emulate osteolytic bone metastases. Taken together, this reproducible and accurate preclinical model, mirroring metastatic bone pain represents a valuable tool to evaluate the therapeutic potential of new analgesic or cancer drug candidates.

### Effect of chronic morphine on tumor progression, skeletal complications and pain relief

Pain is a severe problem in patients copying with bone metastases. Typically, control of bone cancer pain requires escalating doses of morphine or other opioids, which often results to the development of adverse effects, like constipation, tolerance or even opioid-induced hyperalgesia^47^. In the present study, we found that repeated morphine administration was effective at controlling tumor-induced tactile hypersensitivity, probably due to its ability to reduce spinal sensitization, as measured by the decrease in both neuronal and astrocytic activation. Accordingly, acutely or chronically administrated morphine was previously shown to significantly attenuate the pain-related behaviors in tumor-bearing rats^18,48–50^. Here, we further demonstrated using multimodal MRI/TEP imaging and post-mortem µCT that prolonged use of morphine was not effective in delaying or preventing tumor-induced bone loss. More alarming, morphine affected the bone homeostasis by disturbing the healthy balance between bone formation and breakdown. These results are in accordance with previous findings showing that morphine treatment increases the osteoclast activity and the presence of the bone resorption markers, TRAP5b and collagen type-I (CTX) in sera, as well as reduces the levels of serum osteocalcin, a marker of osteoblast activity^17,18^. Likewise, clinical studies also report the damaging effects of opioids on bone health, inducing decreased in bone mineral density (BMD) and increased risk of skeletal fractures in chronic analgesic users^51–55^. These unfavorable effects of morphine on bone homeostasis may be in part due to direct action on osteoblasts and osteoclasts. Indeed, opioid receptors are present on osteoblast-like MG-63 cells and µ-opioid receptor activation by proenkephalin-derived peptides or morphine has been associated with a decrease in alkaline phosphatase activity and osteocalcin synthesis^19,20^. To our knowledge, there is however no evidence of the expression of µ-opioid receptors by osteoclasts. We might therefore hypothesize that morphine enhances osteoclastogenesis through its off-target action at TLR4 receptors. There is indeed a growing literature indicating that morphine may cause pain enhancement via binding and activation of the MD2/TLR4 receptor complex^56,57^. Since TLR4 is also expressed by osteoclasts and its activation by lipopolysaccharide (LPS) promotes bone resorption^58^, we can speculate that the unfavorable effects of morphine on bone strength may be mediated through a TLR4-dependent mechanism. Finally, despite the conflicting literature about the positive or negative action of morphine on tumor growth^13,16,59^ we have examined the effect of morphine on tumor development. We found *in vitro* that morphine did not directly exert tumor growth-promoting or -inhibiting effects on MRMT-1 cancer cells, which was consistent with the absence of µ-opioid receptor expression by those breast carcinoma cells. We cannot however exclude that morphine treatment may indirectly favor bone tumor spread *in vivo* by promoting tumor invasion and angiogenesis^13^, or as found here by weakening of the bone through its action on the osteoblast/osteoclast balance.

### Effect of acute zoledronate on tumor burden, bone remodeling and pain management

Improveme-nts in the survival of breast cancer patients have led to the emergence of bone health as a key aspect of bone pain management and preservation of patient’s quality of life. To date, the nitrogen-containing bisphosphonate zoledronate and the monoclonal antibody denosumab (acting as a RANKL inhibitor) are the main FDA-approved medications prescribed to limit or delay the skeletal-related events in patients copying with bone metastases^21,24^. In the present study, we therefore studied the effects of zoledronate, which avidly binds to the mineralized bone matrix on tumor-induced bone destruction, tumor growth and bone cancer pain. We first observed by combining MRI/TEP imaging and post-mortem µCT that treatment with zoledronate was effective in reducing bone tumor progression and bone remodeling. Indeed, acute zoledronate protected against bone loss by normalizing the tumor-related changes on bone morphometric parameters, including significant increase in bone volume density (BV/TV) and trabecular thickness (Tb.Th), and decrease in trabecular bone pattern factor (Tb.Pf) and porosity. Accordingly, we further found that this improvement in bone strength and architectural integrity was accompanied by a strong inhibitory action of zoledronate on osteoclast activity. These results are consistent with previous findings demonstrating that nitrogen-containing bisphosphonates exert their antiresorptive effects by acting directly on osteoclast function and survival^60^. Bisphosphonates are indeed able to induce apoptosis through specific inhibition of the farnesyl pyrophosphate synthase, a rate-limiting enzyme of the mevalonate pathway involved in the protein prenylation of small GTPases, thereby affecting their intracellular distribution and function in osteoclasts. We also noted at the bone-tumor interface that zoledronate induced a decrease in osteoblast activity *in vivo*. Although the actions of zoledronate on osteoblast function remain controversial, numerous studies have demonstrated that zoledronate negatively affects their proliferation, cell viability and differentiation in a concentration-dependent manner^61–63^. These findings have therefore raised the possibility that the development of osteonecrosis of the jaw might in part be caused by the cytotoxic action of zoledronate on osteoblasts.

Aside of their antiresorptive actions, bisphosphonates have also been described to exert anticancer activity through direct or indirect mechanisms^25,26^. Here, zoledronate did not exhibit *in vitro* anti-proliferative effects against MRMT-1 cancer cells. These results therefore suggest that inhibition of the skeletal tumor burden by zoledronate is mainly driven by indirect antitumor activities. By inhibiting osteoclast-mediated bone resorption, zoledronate may indeed deprive cancer cells of bone-derived growth factors and cytokines, which promote tumor cell growth and survival. Nonetheless, activation of γδT cell cytotoxicity or inhibition of angiogenesis may also represent other potential mechanisms by which zoledronate could exert antitumor effects^25,26^. Finally, we found that the systemic delivery of zoledronate was effective in reversing the pain-related behaviors. While it is widely accepted that bisphosphonates can reduce bone pain by inhibiting osteoclast function, we cannot however exclude a direct central analgesic action of zoledronate. Indeed, bisphosphonates have been found to be effective in managing different types of pain, not related to bone pathologies. Bisphosphonates exert antinociceptive action in acute pain and can prevent mechanical/thermal hypersensitivity associated with the development of neuropathic or inflammatory pain^64,65^. Our results also suggest that zoledronate relieves bone cancer pain, at least in part, through inhibition of neuronal and astrocyte activation, a plastic adaptation that contributes to central sensitization. These results are consistent with previous findings demonstrating that central or peripheral bisphosphonate delivery significantly attenuates bone tumor-induced neuronal activation and reactive gliosis^66^.

### Effect of chronic oral nabilone on tumor growth, bone health and pain control

Metastasis to distant secondary sites is the leading cause of cancer morbidity and mortality in advanced breast cancer patients. Although advances in early detection, radiotherapy and chemotherapy have considerably reduced disease recurrence and death, the major clinical complications associated with cancer-induced bone disease remain the osteolysis associated with enhanced osteoclast activity and the presence of intractable bone pain. In recent years, the endocannabinoid system has emerged as a potential target for the regulation of bone metabolism, tumorigenesis and pain sensitivity^67^. In the present study, we have therefore examined whether the synthetic CB1/CB2 cannabinoid receptor agonist nabilone, which is prescribed as antiemetic or indicated to treat appetite loss in cancer patients undergoing chemotherapy may serve for the treatment of metastatic bone diseases. The combined 3D quantitative µCT analysis and PET/MRI imaging demonstrates here that chronic oral administration of nabilone significantly protects both the cortical and trabecular bone from osteolysis and limits the expansion of bone tumor cells. This increase in bone strength was further accompanied by a re-establishment of the balance between osteoclasts and osteoblasts, thus resembling the effects of zoledronate on bone remodeling. Accordingly, there is accumulating evidence demonstrating that endocannabinoids and their receptors play important roles in skeletal homeostasis by regulating bone cell function and bone turnover^34^. Indeed, mice lacking either of the cannabinoid receptors CB1 or CB2 exhibit abnormal bone phenotypes. Likewise, the pharmacological modulation of the endocannabinoid system influences bone cell activity and bone remodeling in health and disease^33^. For instance, CB2 knockout mice display an osteoporosis-like phenotype and CB2 receptor activation reverses ovariectomy-induced bone loss in wild-type animals by inhibiting the proliferation and activation of osteoclasts^68^. Our results further demonstrated *in vitro* that the CB1/CB2 agonist nabilone exerted a pronounced growth-inhibitory effect on MRMT-1 breast cancer cells. Since CB2 was the only receptor expressed by those mammary carcinoma cells, we can put forward that the antiproliferative effects of nabilone are probably mediated by CB2 receptor activation. In agreement with these findings, CB2-selective agonists were reported *in vitro* to exert antitumor activity in murine 4T1 and 66.1 or human MDA-MB-231 breast cancer cells^69,70^. Interestingly, CB2-selective agonists were also found to reduce the tumor burden *in vivo*, inhibiting the proliferation and invasion of breast cancer cells into the intramedullary femoral cavity as well as the release of proinflammatory mediators^69,71^. Altogether, these results suggest that nabilone may render the bone marrow less hospitable to tumor growth by exerting direct antitumor activity as well as by blunting the osteoclast-osteoblast crosstalk. Further investigation evaluating parathyroid hormone and calcitonin levels or changes to the RANK/RANK-L pathway will be required to fully understand how nabilone, via CB1/CB2 receptors protects the bone from the tumor burden.

In the last decade, the therapeutic use of cannabis-based medicines (herbal, plant-derived, synthetic) has been widely reviewed with a lack of consensus regarding the efficacy of cannabinoid-based therapy in pain medicine. While preclinical studies provide clear evidence of the antinociceptive effects of cannabinoids in various animal models of chronic pain, results from randomized controlled clinical trials do not give active support to prescribing cannabinoids for pain in clinical practice^36,72^. In the preclinical setting, there is also a growing literature indicating that cancer-induced bone pain can be effectively managed by the use of cannabinoids. Indeed, systemic and spinal administration of CB1 (ACEA, PrNMI), CB2 (JWH015, AM1241) and mixed CB1/CB2 (WIN55,212-2) agonists were shown to exert anti-allodynic and anti-hyperalgesic effects in different models of bone cancer pain^69,73–78^. Since the CB1/CB2 agonist nabilone is currently approved to treat the iatrogenic effects of anticancer therapies, we further determined whether its chronic oral administration was effective to attenuate bone cancer pain. To our knowledge, this is the first study investigating the effects of nabilone in the management of bone cancer pain. Our results showed that changes in bone remodeling and tumor growth observed after *per os* nabilone did not translate into a reduction in painful behaviors. The high lipophilicity of this synthetic analog of THC that can lead to poor oral bioavailability may explain its absence of analgesic effects. Moreover, immunohistochemical analysis of c-Fos neuronal and GFAP astrocyte activity within the spinal cord revealed that bi-daily oral administration of nabilone induced a significant decrease in c-Fos activation but no changes in astrocyte activity. Together, these results may highlight the central role played by spinal astrocytes in maintaining chronic pain sensitization^79,80^. In contrast to these findings, clinical studies demonstrated that chronic nabilone may provide small but significant reductions in pain in neuropathic, fibromyalgia and cancer patients^38,81–84^. However, nabilone (dosage range from 0.5–4 mg/kg per day) was used as an adjunctive treatment in the vast majority of these studies^37^. Here, we opted for a therapeutic dose of 1 mg/kg per day of nabilone to avoid potential adverse effects, such as drowsiness. Despite the fact that this dose was effective at reducing the skeletal tumor burden and at limiting bone resorption *in vivo*, optimal doses have yet to be determined to counteract bone cancer-induced pain symptoms.

## Conclusions

In conclusion, longer survival with metastatic breast cancer increases the need of developing better treatment options to preserve or improve patient’s quality of life and well-being. In particular, these bone cancer pain management strategies need to consider the fact that tumor burden, bone damage and pain are interrelated. Here, we report that chronic use of morphine, typically prescribed as pain reliever may have detrimental effects on bone healing, thus inviting practitioners to closely monitor morphine-treated patients, notably those with a history of osteoporosis. With the recent progress in approving cannabinoid-based therapy for medical indications and even recreational use, it becomes more and more important to determine their health benefits and risks of adverse drug reactions. Our results demonstrate that chronic oral administration of a low dose of nabilone may improve bone health and reduce tumor proliferation. Based on these findings, it is therefore tempting to propose the use of nabilone as an adjuvant therapy to conventional treatment in order to address the multifaceted nature of bone cancer pain. In favor of this polypharmacy intervention, recent studies proposed that cannabinoids, including nabilone may have opioid-sparing effects or synergistic effects with opioids^38,39,85^. Finally, it is also important to mention that nabilone can also reduce the burden of sleep disturbances and anxiety disorders, which are both common problems in patients living with metastatic breast cancer^37^.

## Materials and Methods

### Animals

Female Sprague-Dawley rats (150–175 g) were maintained on a 12 h light/dark cycle with access to food and water *ad libitum*. All animal procedures were approved by the ethical committee for animal care of the *Université de Sherbrooke*, in compliance with the policies and directives of the *Canadian Council on Animal Care*.

### Cell culture

MRMT-1 rat breast carcinoma cells were kindly provided by the *Cell Resource Center for Biomedical Research Institute of Development, Aging and Cancer* (Tohoku University) and were harvested in RPMI 1640 medium supplemented with 10% FBS and 2% penicillin/streptomycin.

### MTT assay

For cell viability assay, 15,000 cells were seeded in triplicate in a 96-well plate in RPMI 1640 medium without phenol red supplemented with 10% FBS and 2% penicillin/streptomycin. The assay is based on the reduction of the yellow soluble MTT reagent 3-[4,5-dimethylthiazol-2-yl]-2,5-diphenyl tetrazolium bromide into an insoluble formazan product measurable at 540 nm. 12 h after cell seeding, 100 µl of fresh serum- and phenol red-free medium containing from 10^−10^ M to 10^−4^ M of either morphine sulfate, zoledronate (both diluted in PBS), nabilone (0.4% DMSO), 5 to 40 µM tamoxifen (0.07% ethanol) or the corresponding vehicle was added. After 24 h, 48 h and 72 h drug incubation, 10 µl of 5 mg/ml MTT reagent was added to each well and incubated for 4 h at 37 °C, 5% CO_2_. Cells were centrifuged at 1,000 × g for 5 min. and the medium was carefully removed. 100 µl DMSO was added to dissolve the formazan crystals and the optical density (OD) of each well was measured at 540 nm using a Molecular Devices ThermoMax Microplate Reader. Cell viability was measured using the following formula: (OD_540nm_ treatment/OD_540nm_ vehicle) × 100.

### Quantitative RT-PCR

MRMT-1 cell mRNA were extracted using RNeasy Mini Kit following the manufacturer’s protocol. RNA integrity was assessed using an Agilent 2100 Bioanalyzer. RNA quality and presence of contaminating genomic DNA was verified as previously described^86^. Reverse transcription was performed using 1 µg total RNA with QuantiTect reverse transcriptase, random hexamers and dNTPs in a total volume of 20 µl. All forward and reverse primers were individually resuspended to 20–100 μM stock solution in Tris-EDTA buffer and diluted as a primer pair to 1 μM in RNase DNase-free water. qPCR reactions were performed in a volume of 10 µl in 96 well plates on a CFX96 Thermal Cycler with 5 μL of 2X iTaq Universal SYBR Green Supermix, 10 ng cDNA and 200 nM primer pair solution. The following cycling conditions were used: 3 min at 95 °C; 50 cycles, 15 s at 95 °C, 30 s at 60 °C, 30 s at 72 °C. Primer design and validation were evaluated as described elsewhere^86^. All primer sequences are available in Table 1. The amplified products were analyzed by automated chip-based microcapillary electrophoresis on a Caliper LabChip 90 instrument. Amplicon sizing and relative quantitation was performed by the manufacturer’s software, before being uploaded to the LIMS database.

**Table 1:** Primer sequences used for quantitative real-time RT-PCR analysis.

Gene	Forward Sequence	Reverse Sequence
Cnr1 (NM_012784.5)	GGAGAACATCCAGTGTGGGG	CATTGGGGCTGTCTTTACGG
Cnr2.1 (NM_001164143.3)	CTCCTGGGCTGGCTTCTTTTCATT	CTCTCCACTCCGCAGGGCATAA
Cnr2.2 (NM_001164142.3)	CGAGGCCACCCAGCAAACAT	GGGTTGAACTCCAAGCCGCCA
Oprm1 (NM_013071.2)	GCCATCGGTCTGCCTGTAAT	GAGCAGGTTCTCCCAGTAC
Oprd1 (NM_012617.1)	CCCAGTGCGAGCGCC	GGTGGCCGTCTTCAG

### Surgery

All rats were randomly assigned either to cancer or sham surgery groups. Syngeneic MRMT-1 breast cancer cells were surgically implanted as described by Doré-Savard *et al*.^8–10^. Briefly, 30 000 cells were diluted in 20 µL Hank’s Balanced Salt Solution (HBSS) and injected into the medullary cavity of the female rat femur after a minimal opening by a microdrill. The hole was then sealed with dental amalgam. During the surgery, rats were maintained under anesthesia using isoflurane 5%, 2% O_2_ for induction followed by 2.5% isoflurane and 1.5% O_2_. No post-operative analgesia was used to avoid interference with pain assessment. Saline-injected rats are tumor-bearing rats treated with the vehicle solution whereas sham-operated female rats received the surgery, but no cancer cells were delivered to bone marrow in the femur. Sham-operated rats also received saline.

### Drug administration

Eleven days after cancer cell implantation surgery, animals were randomly assigned to control or treatment group. Rats received bi-daily administrations of either morphine sulfate (6 mg/kg/day in saline, subcutaneous) or nabilone (1 mg/kg/day in Syrup BP, *per os*, RAN-Nabilone DIN 02358093) or their corresponding vehicles between day 11 until day 18. A zoledronate acute administration group was also included in our investigation as an inhibitor of bone resorption. According to the pharmacokinetic profile of zoledronate and the monthly dosing in patients with bone metastases^87^, we performed a single subcutaneous administration on day 11 (0.1 µg/rat in PBS, pH 7.4). Zoledronate-treated rats further received bi-daily subcutaneous saline injections to control for manipulation-induced stress. All injections were performed one hour prior to behavioral testing.

### Behavioral studies

Mechanical sensitivity was assessed using an automatic *von Frey* dynamic plantar æsthesiometer by an experimenter blinded to treatment. Rats were individually placed in clear plexiglass boxes over a wired mesh floor. The von Frey filament was applied under the hind paw plantar surface of the rat and exerted a linear increasing pressure (3.33 g/s). Stimulations were automatically stopped by animal’s paw withdrawal response or when the 50 g cut-off was reached. Measures were repeated five times alternately on each hind paw.

### MRI imaging

MRI studies were conducted at the *Centre d’Imagerie Moléculaire de Sherbrooke* (CIMS) with a 210 mm diameter small-animal 7T scanner and a 63 mm diameter volume RF coil. Anaesthetized rats were placed supine in an MRI-compatible cradle equipped with a custom-made paw support designed to position limbs both stably and reproducibly. The MRI protocol included the acquisition of sagittal Proton Density-weighted images using a fast spin-echo pulse sequence with a repetition time of 3000 ms and with an effective echo time of 10 ms, 8 echoes and 8 averages. We acquired 30 sagittal slices of 30 µm of thickness with a field of view of 60 × 60 mm in a matrix of 256 × 256 resulting a final resolution of 23.47 × 23.47 × 30 µm^3^.

### PET imaging

After MRI acquisition, the cradle was transferred to a PET scanner without interrupting the anesthesia or animal monitoring. PET imaging was performed using a LabPET4 (Gamma Medica) avalanche photodiode detector-based small-animal PET scanner with a field of view of 110 mm in diameter by 37.5 mm in axial length. The scanner achieves a spatial isotropic resolution of 1.35 mm in full width at half maximum and an absolute sensitivity of 1.1% in the central field of view, with a 250 to 650 keV energy window. Rats were aligned to have the hind knee joints at the radial and axial center of the scanner field of view, and they received approximately 37 MBq of ^18^Fluoro-deoxyglucose (^18^F-FDG) by intravenous injection (200 μL at 500 μL/min). 30 min after ^18^F-FDG administration, the accumulation of radiotracers in the target tissues was monitored by 30-min static imaging. Images were reconstructed using the Triumph PET/CT software implemented with a 3D-MLEM algorithm using 20 iterations, span of 63, field of view of 80 mm with a final matrix resolution of 160 × 160 × 128 and a voxel size of 0.5 × 0.5 × 0.597 mm^3^.

### Image visualization and analysis

PET and MR images were fused using a multi-resolution binarized intensity histogram (MRBIH) co-registration procedure that was specifically developed for small-animal PET/MR image fusion^8^. The procedure combined low- and high-resolution images to take advantage of the low noise sensitivity at coarse levels and higher contrast at higher levels. MR imaging was used as fixed volume, and PET was used as the floating volume. We used three multi-resolution levels for each MRBIH and aligned them for nine parameters (translation, rotation, and scaling in axial, sagittal, and coronal planes) until they converged at the same set of parameters. Co-registered PET/MR images were visualized and analyzed with an OsiriX viewer (version 4.0, 64 bit; OsiriX). To determine the level of variation in radiotracer uptake, the mean standardized uptake values (SUV_mean_) were calculated according to the following formulae:

$$SU{V}_{mean}=mean\,uptake\,value/(dose\,injected[MBq]\times animal\,weight[kg])$$

The SUV_mean_ were valued within the boundaries of a region of interest (ROI), which was delimited on the MR images of the cancer-implanted paw. For each T_1-weigthed_ image, the longitudinal plan of the femur was identified, and a mask of the femur has been manually drawn to assess the effect of the pharmacological regimen on tumor growth. ROIs were drawn to define the diaphysis (shaft) and the distal metaphysis or epiphysis (extremity) of the femur. As the metastasis grew into the bone after implantation, we included the tumoral mass within the mask. The ROIs were then pasted on the corresponding co-registered PET images. Raw data were extracted from these ROIs, and the SUV_mean_ and femur area were calculated. ROIs were drawn on three consecutive slices per paw.

### *Ex vivo* µCT

Anesthetized rats were intra-aortically perfused with 500 ml 4% paraformaldehyde solution. Ipsilateral femurs were removed and post-fixated 48 h in the same solution then washed in PBS for *ex vivo* µCT experiment. Scans were performed at the *McGill Bone Center* using a high-resolution desktop Micro-CT scanner. Rat femurs were scanned at X-ray source power of 45 keV/222 µA and at a resolution of 11.25 µm/pixel. The µCT images were reconstructed using NRecon (v1.6.1.3) and CT-Analyzer (v1.10.0.2) provided by SkyScan which was used for reconstruction and 3D analyses, respectively. The Volume of Interest (VOI) of cortical + trabecular bone is defined as the total (tissue) volume including cortical bones, trabecular bones and any spaces over the range of 5.626 mm (201 cross sections) starting from the growth plate in the distal femur. VOI for trabecular is defined as the total (tissue) volume including all trabecular bones and any spaces over the range of 5.626 mm (201 cross sections) starting from the growth plate in the distal femur.

### Bone histology and immunolabeling

After µCT scanning, femurs were embedded in a mixture of 50% methyl methacrylate (MMA) and 50% glycolmethacrylate (GMA). Then, serial 6 μm sections of embedded tissues were generated to be stained either with Hematoxylin and Eosin or toluidine blue, or to measure the tartrate-resistant acid phosphatase (TRAP) and alkaline phosphatase (ALP) activities. ALP-positive osteoblasts were observed by staining with Naphthol AS-TR phosphate, N,N-dimethyl formamide, and nitroblue tetrazolium/bromochloroindolyl phosphate in tris-malate buffer (pH 9.3). Osteoclasts and other mononuclear TRAP-positive cells were observed by staining with Naphthol AS-TR phosphate, sodium nitrite, sodium tartrate, and pararosaniline hydrochloride in acetate buffer (pH 5.0). A set of 10 images for each animal has been acquired. From each image, the boundary between the cortical bone and the bone marrow was manually delineated and each nucleus of ALP- or TRAP-positive cells along the cortical line was identified and counted. Only polynuclear osteoclasts fixed to the cortical line were counted and included in this analysis. This results in a number of osteoblasts (ALP) or osteoclasts (TRAP) per square millimeter for each image and the average of all images was calculated to evaluate the overall activity of osteoblasts and osteoclasts. Osteoblast and osteoclast counts were obtained using Bioquant Nova Prime image analysis software. Femur immunohistochemistry was performed in longitudinal, unstained and calcified sections to avoid epitope degradation induced by EDTA solution. MMA-GMA was removed by two 30 min slice immersions in 2-methoxylethyl acetate, then plastic was removed from the samples in two 5 min baths of xylene and samples were rehydrated in successive baths of decreasing degree of ethanol. Thereafter, samples were equilibrated in Tris-Buffered Saline and 0.1% Tween (TBST) during 30 min and epitope demasking was performed in 10 mM citrate buffer pH 6.0 at 100 °C during 20 min. Slices were then treated in 3% H_2_O_2_ solution for 10 min and blocked in 2% bovine serum albumin (BSA) in TBST for 1 h. Section were incubated at 4 °C overnight with the primary antibody against Ki-67 (1:500, rabbit anti-Ki-67 [SP6]-AB16667, Abcam, ON, CA), ERα (1:200, mouse anti-ERα MA1-310, Pierce, ON, CA) or human epidermal growth factor receptor-2 (HER-2) (1:500, rabbit anti-ErbB2, AB2428, Abcam ON, CA) diluted in blocking buffer. Sections were rinsed in TBST and revealed with corresponding biotinylated goat anti-rabbit (1:500, Vector Labs, Cat number BA-1000, ON, CA) or goat anti-mouse antibodies (1:500, Vector Labs, Cat number BA-9200, ON, CA) and then incubated in Elite ABC solution (Vector Laboratories). The product of immune reaction was revealed using 3,3′-diaminobenzidine (DAB) as a chromogen and 0.015% H_2_O_2_. Finally, slices were dehydrated in graded ethanol, defatted in xylene and mounted with Permount. Immunostaining images were acquired using a Leica DM4000 microscope equipped with a Leica DFC350FX camera using the same acquisition parameters. Human breast cancer HER-2 positive specimens were generously and anonymously provided by the anatomopathology service of the *Centre Hospitalier Universitaire de Sherbrooke* to be used as positive control for HER-2 immunostaining.

### Spinal cord immunostaining

Rats were intra-aortically perfused with 500 mL of freshly prepared 4% paraformaldehyde solution. L1 to L3 spinal cord sections were collected, post-fixed in 4% paraformaldehyde solution at 4 °C for 24 h and then cryoprotected in 30% sucrose solution in 0.1 M PBS at 4 °C for 48 h. Frozen tissue were embedded at −35 °C in O.C.T. compound and 30 µM transverse sections were generated using a Leica SM220R sliding microtome. Free-floating sections were then washed in PBS, blocked in 0.2% Triton X-100 supplemented with 5% normal goat serum and 2% BSA in 0.1 M PBS for 1 h at room temperature. After 20 min incubation in 0.1 M glycine solution, sections were labeled with anti-GFAP antibody (1:1000, chicken anti-GFAP, Chemicon, Millipore, Cat. number AB5541 ON, CA) diluted in blocking solution. Sections were then rinsed twice and incubated with the fluorescent secondary antibody (1:200, AlexaFluor 594 conjugate, goat anti-chicken, Invitrogen, A-11042, ON, CA) in blocking solution for 1 h, washed twice in PBS and mounted on SuperFost Plus slides with Aqua-Poly/Mount. Spinal cord slices were also stained against the c-Fos protein. Briefly, sections were treated in 3% H_2_O_2_ solution for 10 min and then blocked in 2% BSA, 0.1% Triton X-100 in 0.1 M PBS for 1 h at room temperature prior to incubation with c-Fos primary antibody (rabbit anti-c-Fos, 1:5000, Abcam, cat#7963, ON, CA). Slices were rinsed and incubated with biotinylated goat anti-rabbit antibody (1:200, Goat anti-rabbit, Vector Labs, Cat number BA-1000, ON, CA) and then incubated in Elite ABC solution. The product of immune reaction was revealed using 3,3′-diaminobenzidine as a chromogen and 0.015% H_2_O_2_. Sections were mounted on SuperFost Plus slides, dehydrated in graded ethanol, defatted in xylene and mounted with Permount. Fluorescence images of spinal cord slices were acquired at 5x using a Leica DM4000 microscope equipped with a Leica DFC350FX using the same acquisition parameters. GFAP quantification was performed using ImageJ software. GFAP immunofluorescence intensity was quantified in four regions of interest on the ipsilateral side of the spinal cord – the marginal nucleus (lamina I), the substantia gelatinosa (lamina IIo, IIi), the nucleus proprius (lamina III and IV) and the deep lamina VI and VIII according to the cytoarchitectonic organization of the spinal cord (Molander *et al*., 1984). Briefly, acquired images were converted to 8-bit resolution and regions of interest were manually drawn to delimitate each spinal lamina. Ten sections located between L1 and L3 vertebrae were analyzed per animal, for a total of tree animals per treatment condition. For c-Fos immunoreactive nuclei quantification, positive neurons were counted manually on live images using a bright-field microscope and an InfinityX camera by an observer blinded to the treatment conditions. C-fos positive neurons were counted in spinal lamina 7 on the ipsilateral side which was delineated according to Molander. Neurons were considered positive if the nucleus showed the characteristic staining of oxidized DAB and was distinct from the background. Ten sections located between L1 and L3 vertebrae were counted per animal, for a total of tree animals per treatment condition.

### Statistical analysis

All data were collected, compiled and analyzed using GraphPad Prism 6.02 software. Data are presented as means ± standard error of the mean (S.E.M.). Differences between groups were assessed using a One-Way ANOVA followed by Dunnett’s multiple comparisons test. Stars represent differences with the sham group, *p* < 0.05 (*), *p* < 0.01 (**), *p* < 0.001 (***) and hashtags represent differences with the saline group, *p* < 0.05 (#), *p* < 0.01 (##), *p* < 0.001 (###).

## Supplementary information

S1.
