# Publisher Correction: Dichotomic effects of clinically used drugs on tumor growth, bone remodeling and pain management

**DOI:** 10.1038/s41598-020-59781-y

**Published:** 2020-02-14

**Authors:** David André Barrière, Élora Midavaine, Louis Doré-Savard, Karyn Kirby, Luc Tremblay, Jean-François Beaudoin, Nicolas Beaudet, Jean-Michel Longpré, Roger Lecomte, Martin Lepage, Philippe Sarret

**Affiliations:** 1 Département de Pharmacologie-Physiologie/Institut de Pharmacologie de Sherbrooke, Université de Sherbrooke, Québec, Canada; 2 Département de médecine nucléaire et de radiobiologie, Université de Sherbrooke et Centre d’imagerie moléculaire de Sherbrooke, Sherbrooke, Québec, Canada; 3 Département d’Anesthésiologie, Université de Sherbrooke, Québec, Canada

Correction to: *Scientific Reports* 10.1038/s41598-019-56622-5, published online 27 December 2019

In the original version of this Article, the author David André Barrière was incorrectly indexed. This error has now been corrected.

