# Supplementary material for: Dichotomic effects of clinically used drugs on tumor growth, bone remodeling and pain management
Source: Sci Rep. 2019 Dec 27;9:20155. doi: 10.1038/s41598-019-56622-5 (PMC6934511; doi:10.1038/s41598-019-56622-5)
Supplement: Supplementary file 1 — S1. [file 41598_2019_56622_MOESM1_ESM.docx]

**SUPPLEMENTAL INFORMATION FOR**

**Dichotomic effects of clinically used drugs on tumor growth, bone remodeling and pain management**

David André Barrière^1*^, Élora Midavaine^1*^, Louis Doré-Savard^1^, Karyn Kirby^1^, Luc Tremblay^2^, Jean-François Beaudoin^2^, Nicolas Beaudet^1^, Jean-Michel Longpré^1^, Roger Lecomte^2^, Martin Lepage^2^ and Philippe Sarret^1#^

**Table of contents:**

**1. Tumor cell growth assessed using the MTT cell viability assay**


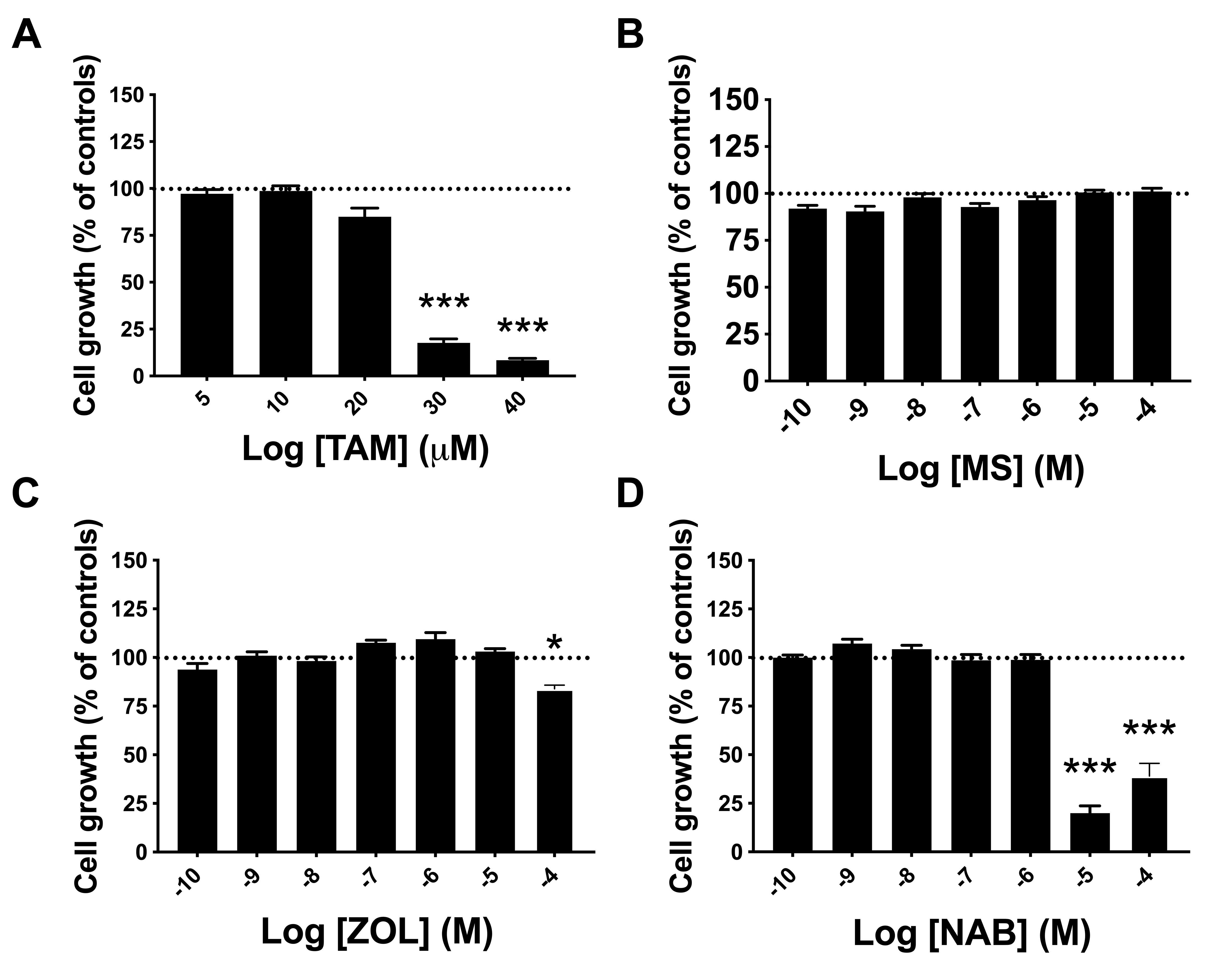


**Figure S1**. *In vitro* dose-concentration of MRMT-1 cell viability upon 24h of treatment with (**A**) tamoxifen, (**B**) morphine sulfate, (**C**) zoledronate or (**D**) nabilone. Tamoxifen, nabilone and zoledronate (at only very high dose) reduce MRMT-1 cell viability while treatment with morphine sulfate fails to achieve such effect at all doses tested. Bars represent mean ± SEM, *n*=3. **p<*0.05, ****p<*0.001 compared to control.

**2. Validation of the CNR1, CNR2 v1, CNR2 v2, Oprm1, and Oprd1 primers used to evaluate respectively the expression of the CB1, CB2 variants, MOR and DOR transcripts in the MRMT-1 cancer line.**

**
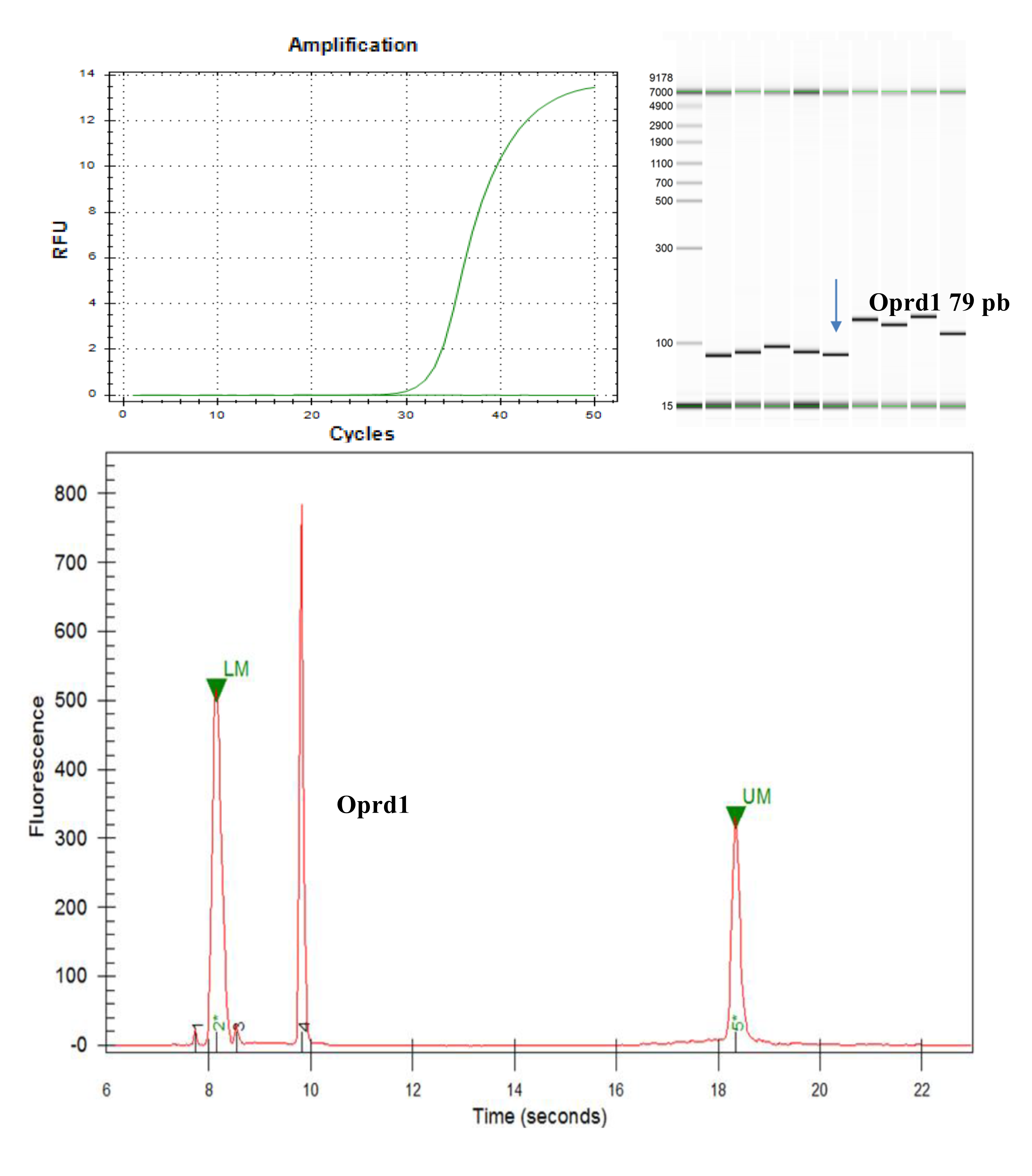
**


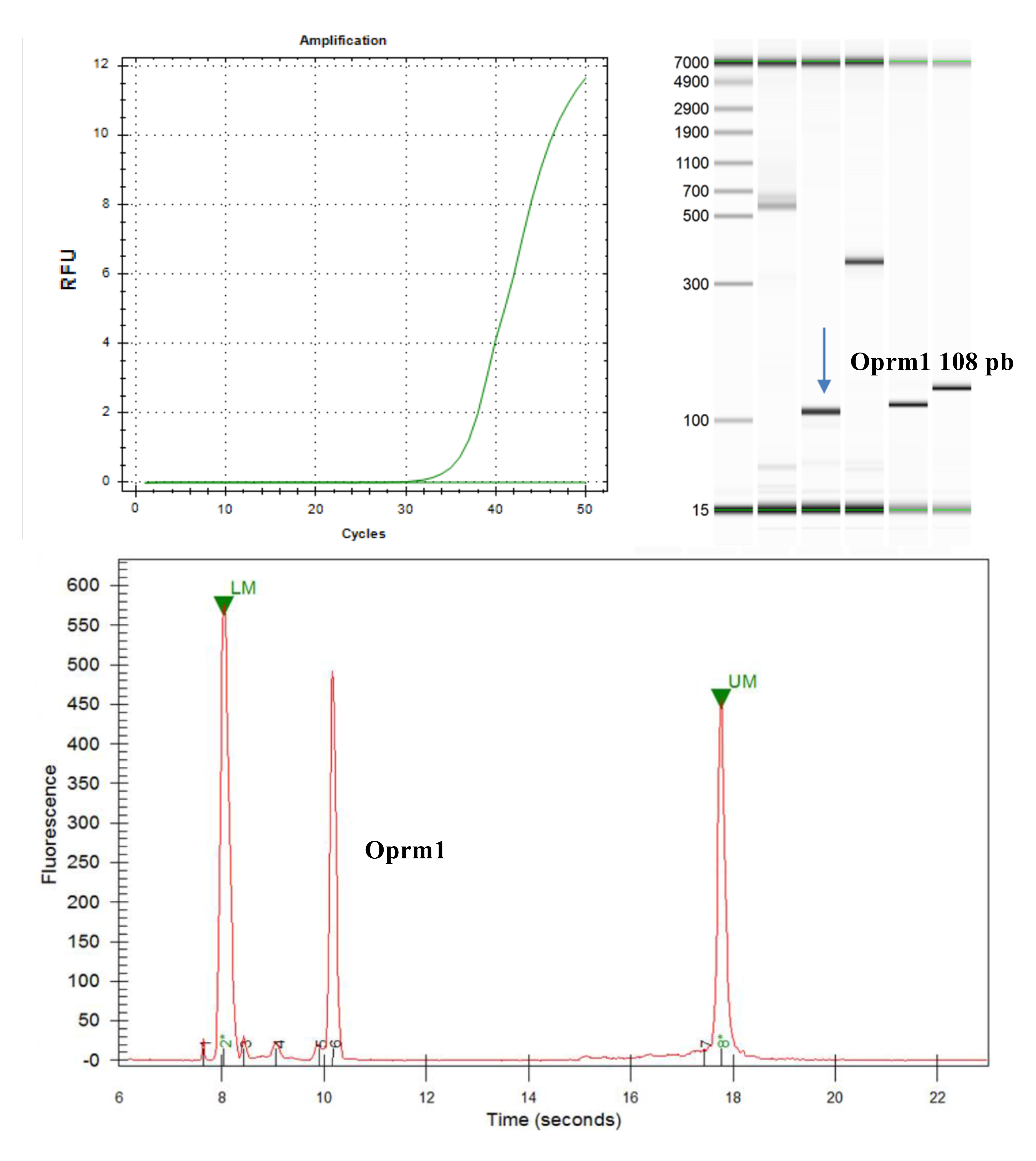


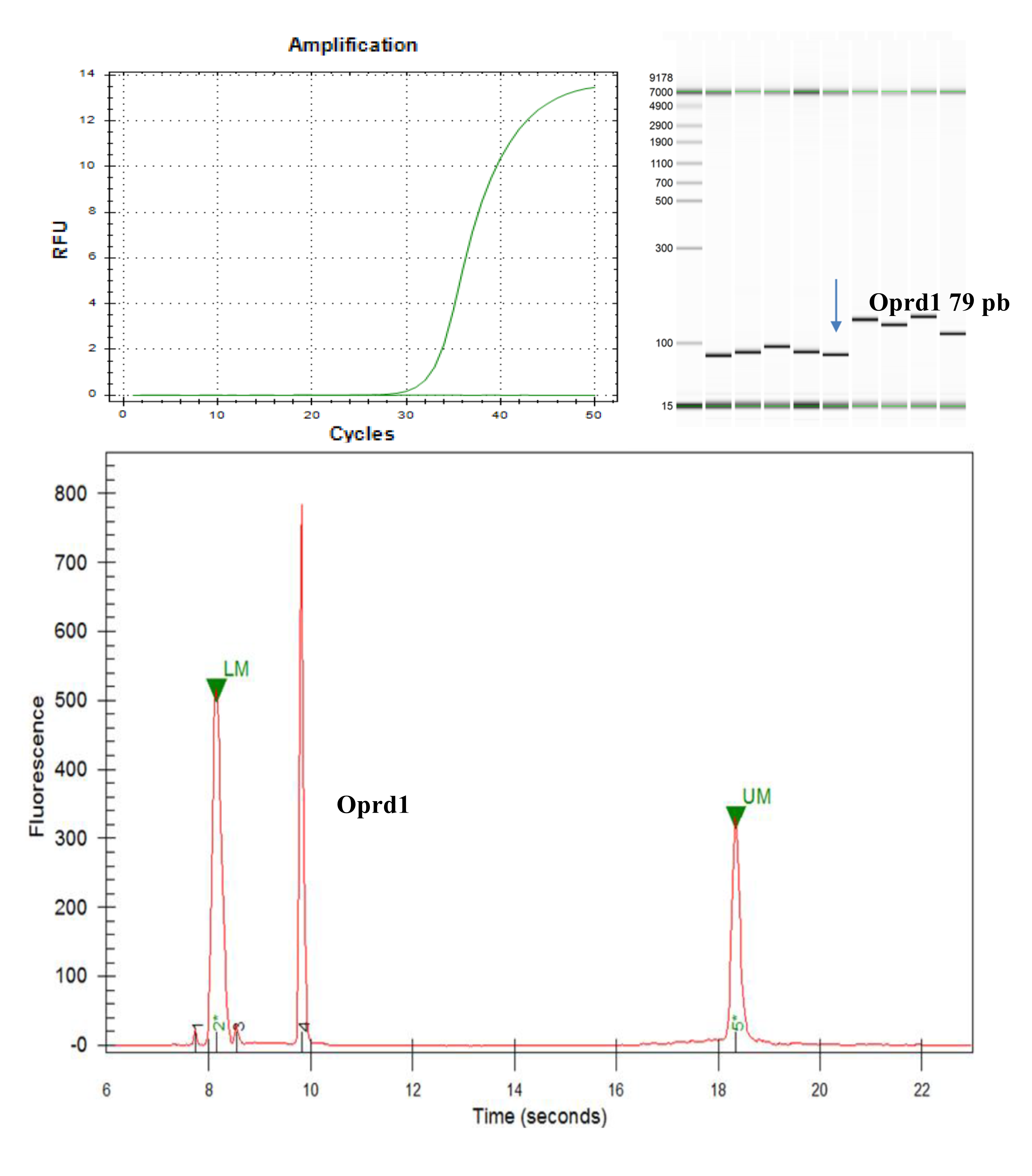

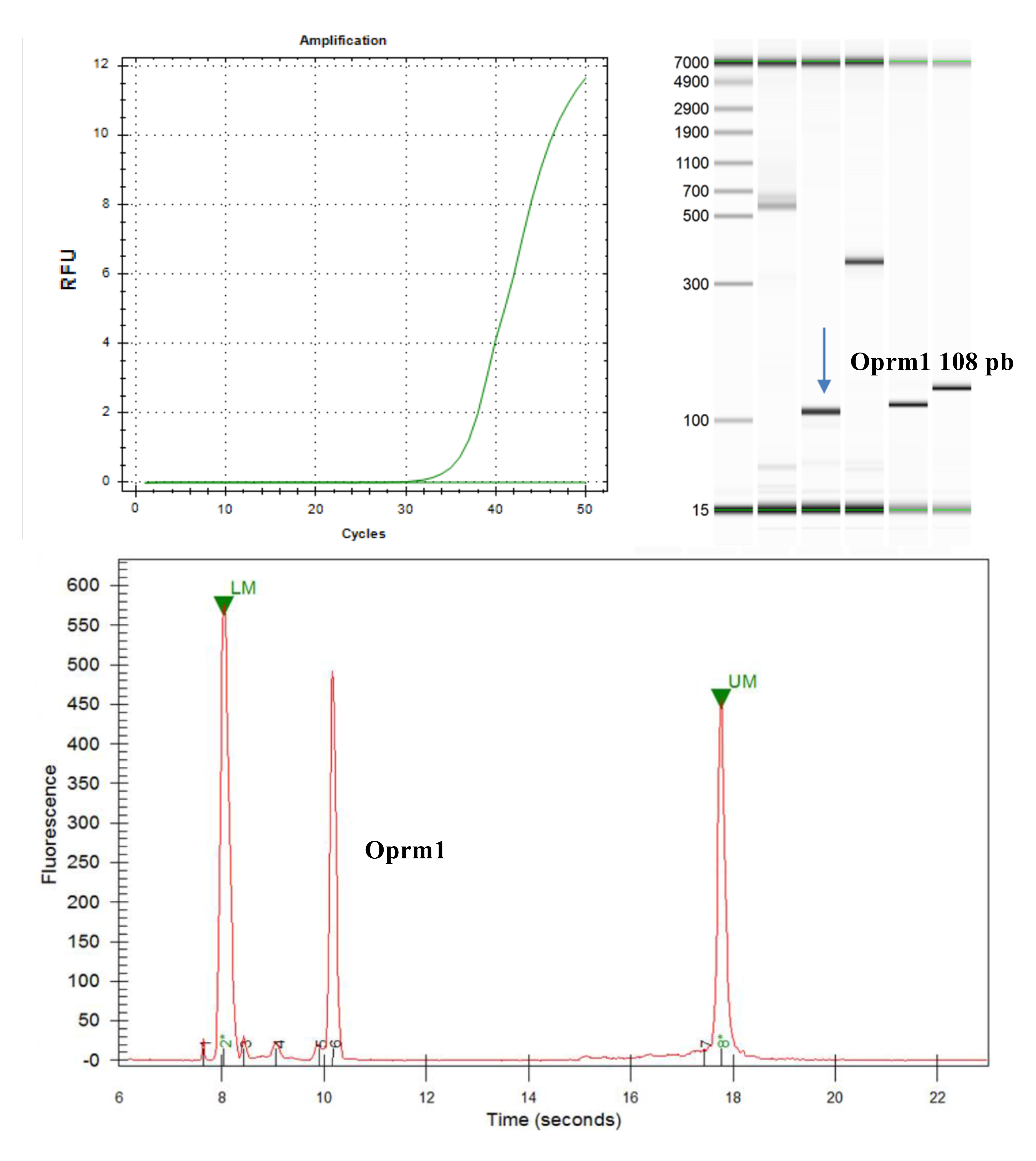


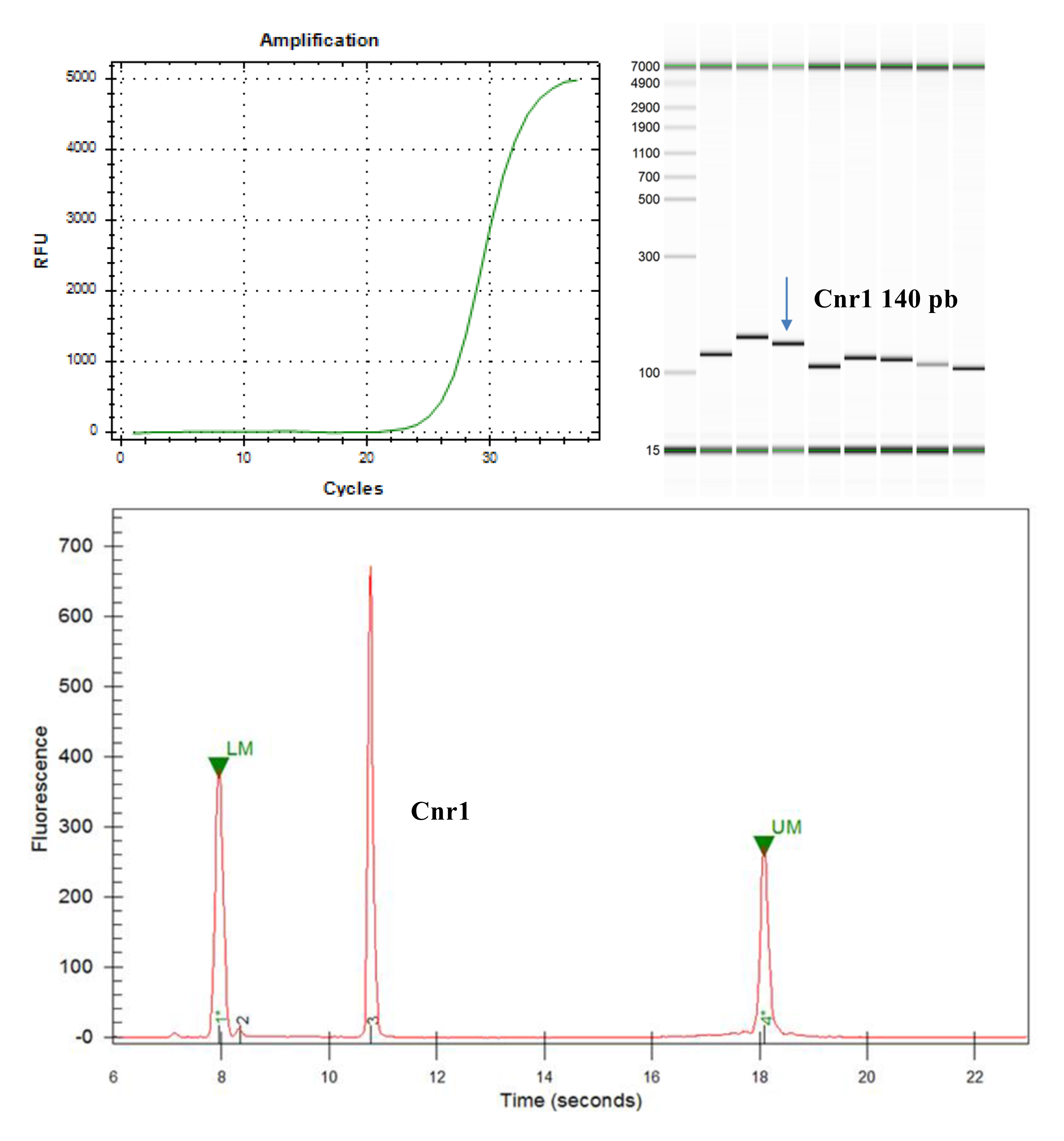


**
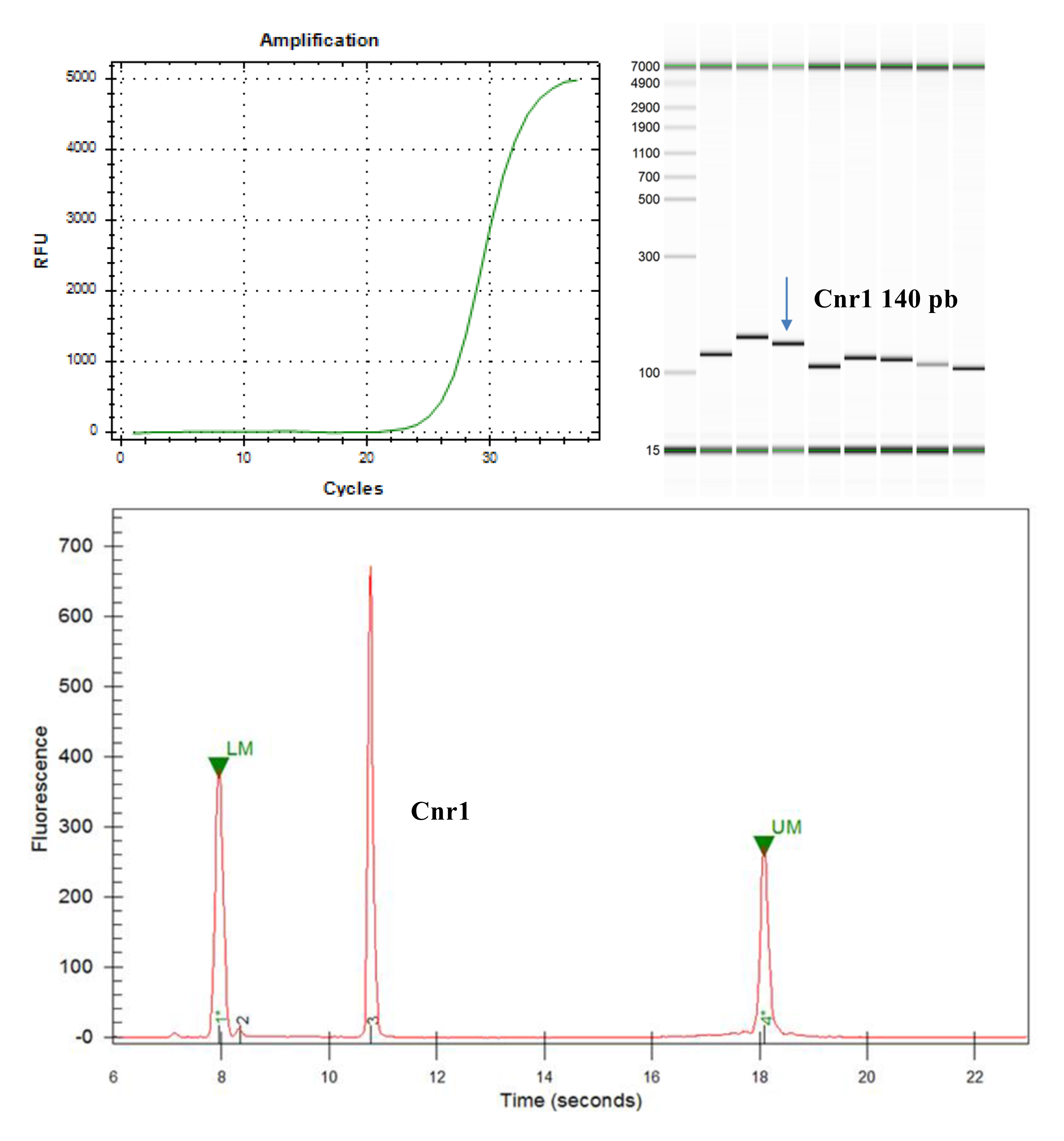
**
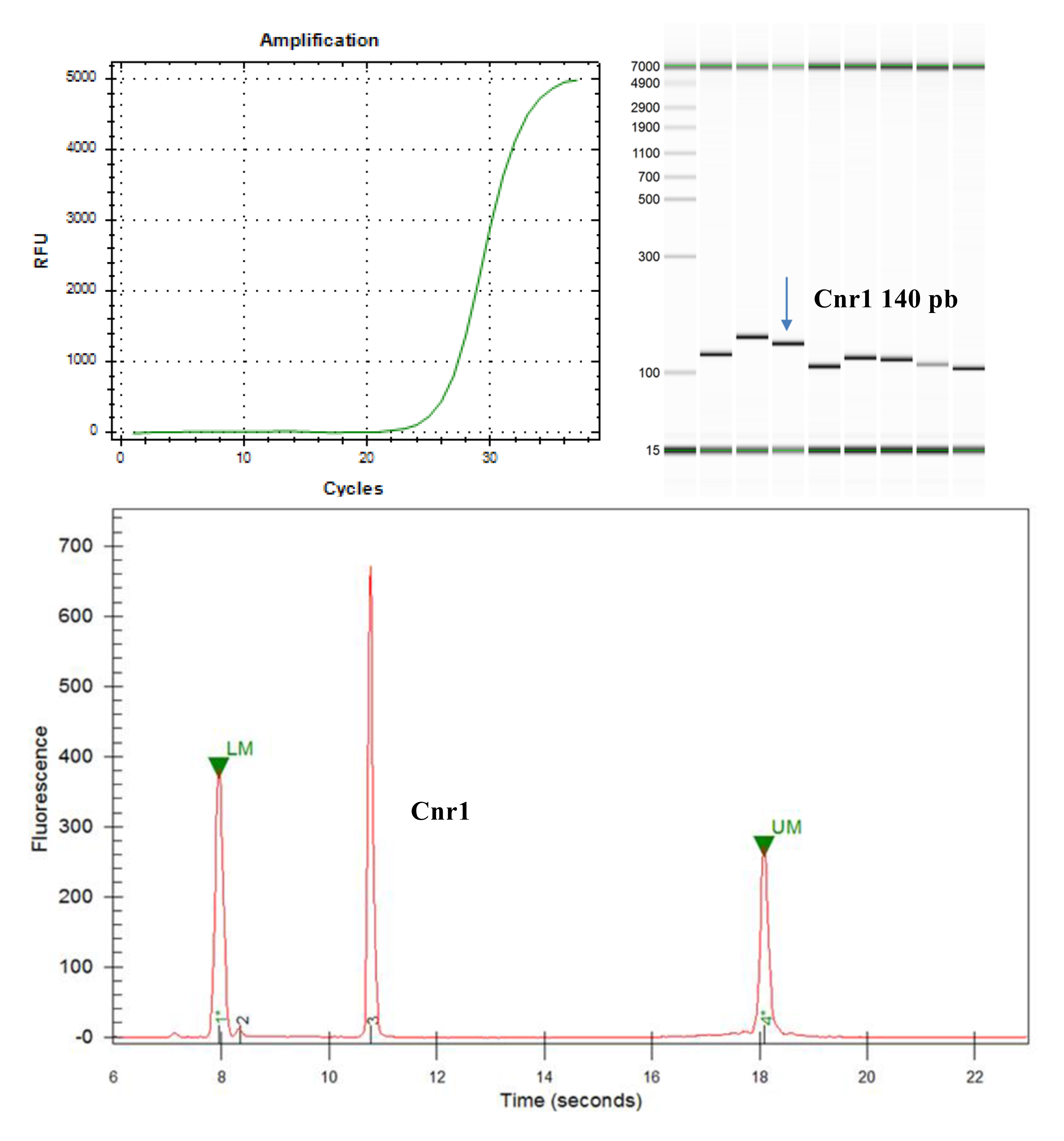


**
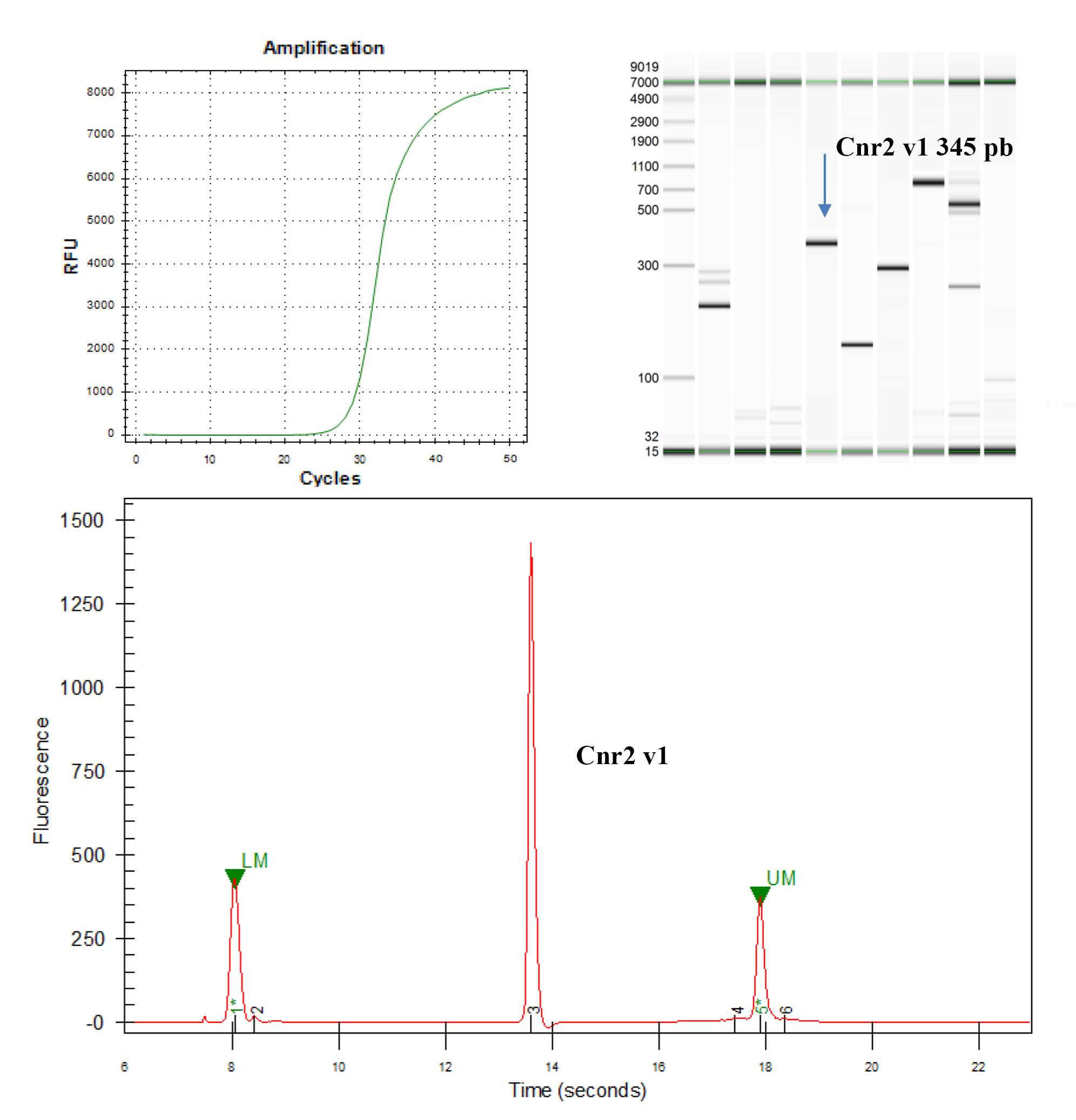
**

**
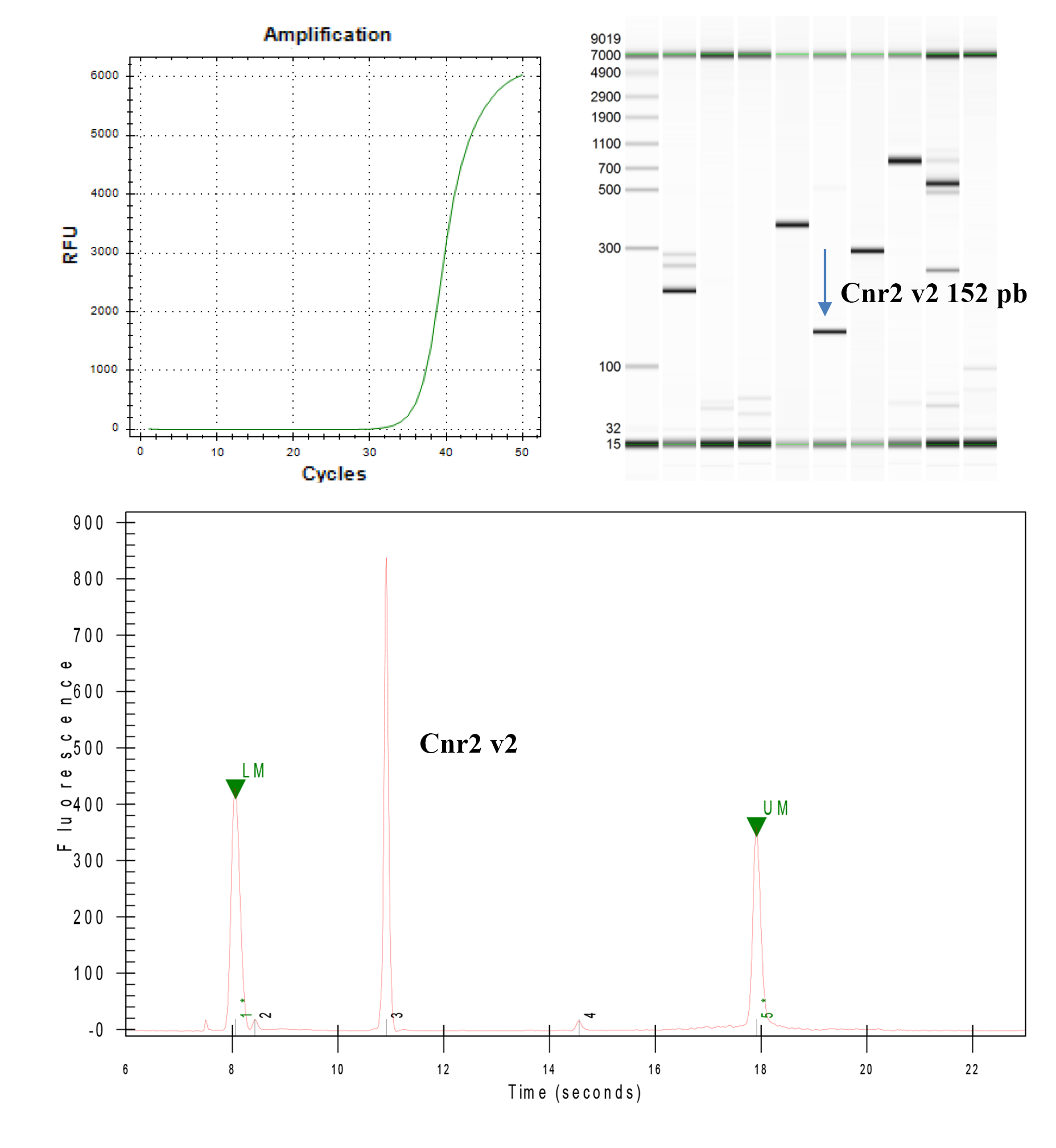
**

**Figure S2**. Assessment of primer efficiency against µ-opioid receptor (Oprm1), δ-opioid receptor (Opdr1) and cannabinoid receptor 1 (Cnr1). Each primer pairs were validated using Rat Universal cDNA. **A**) RT-qPCR amplifications reactions were performed in 96 well plates on a CFX-96 thermocycler using SYBR Green fluorescence and curves are represented as Ct (cycle threshold). Amplification products were analyzed by automated chip-based microcapillary electrophoresis on Caliper LC-90 instruments and represented as **B**) molecular weight in pair base and **C**) fluorescence intensity relative to internal standard markers. Amplification products are identified by a blue arrow with expected molecular weight. RFU: Relative Fluorescence Intensity; LM: internal lower standard marker; UM: internal upper standard marker.
